# Dissecting the Within-Africa Ancestry of Populations of African Descent in the Americas

**DOI:** 10.1371/journal.pone.0014495

**Published:** 2011-01-06

**Authors:** Klara Stefflova, Matthew C. Dulik, Jill S. Barnholtz-Sloan, Athma A. Pai, Amy H. Walker, Timothy R. Rebbeck

**Affiliations:** 1 Department of Biostatistics and Epidemiology, School of Medicine, University of Pennsylvania, Philadelphia, Pennsylvania, United States of America; 2 Department of Anthropology, University of Pennsylvania, Philadelphia, Pennsylvania, United States of America; 3 Case Comprehensive Cancer Center, Case Western Reserve University School of Medicine, Cleveland, Ohio, United States of America; 4 Department of Human Genetics, University of Chicago, Chicago, Illinois, United States of America; 5 Abramson Cancer Center, School of Medicine, University of Pennsylvania, Philadelphia, Pennsylvania, United States of America; University of Otago, New Zealand

## Abstract

**Background:**

The ancestry of African-descended Americans is known to be drawn from three distinct populations: African, European, and Native American. While many studies consider this *continental* admixture, few account for the genetically distinct sources of ancestry within Africa – the continent with the highest genetic variation. Here, we dissect the *within-Africa* genetic ancestry of various populations of the Americas self-identified as having primarily African ancestry using uniparentally inherited mitochondrial DNA.

**Methods and Principal Findings:**

We first confirmed that our results obtained using uniparentally-derived group admixture estimates are correlated with the average autosomal-derived individual admixture estimates (hence are relevant to genomic ancestry) by assessing *continental* admixture using both types of markers (mtDNA and Y-chromosome vs. ancestry informative markers). We then focused on the *within-Africa* maternal ancestry, mining our comprehensive database of published mtDNA variation (∼5800 individuals from 143 African populations) that helped us thoroughly dissect the African mtDNA pool. Using this well-defined African mtDNA variation, we quantified the relative contributions of maternal genetic ancestry from multiple W/WC/SW/SE (West to South East) African populations to the different pools of today's African-descended Americans of North and South America and the Caribbean.

**Conclusions:**

Our analysis revealed that both *continental* admixture and *within-Africa* admixture may be critical to achieving an adequate understanding of the ancestry of African-descended Americans. While *continental* ancestry reflects gender-specific admixture processes influenced by different socio-historical practices in the Americas, the *within-Africa* maternal ancestry reflects the diverse colonial histories of the slave trade. We have confirmed that there is a genetic thread connecting Africa and the Americas, where each colonial system supplied their colonies in the Americas with slaves from African colonies they controlled or that were available for them at the time. This historical connection is reflected in different relative contributions from populations of W/WC/SW/SE Africa to geographically distinct Africa-derived populations of the Americas, adding to the complexity of genomic ancestry in groups ostensibly united by the same demographic label.

## Introduction

The ancestry of people in the Americas self-identified as having origin in Africa reflects the relatively recent admixture of three “continental” ancestral populations: African, European, and Native American [1]. This recent admixture has implications for research in population genetics, anthropology, and epidemiology. For example, of anthropological interest is the influence of admixture from displaced Africans on populations in the Americas [2], [3] and gender-specific admixture processes [4]–[7]. In the field of molecular epidemiology, admixture presents a challenge to association studies that could suffer from bias due to confounding by admixture or population stratification [8]–[12].

Typically, association studies use autosomal ancestry-informative markers (AIMs) to correct for population stratification, assessing *continental* admixture by estimating ancestral proportions of an individual's (West) African, European and sometimes also Native American ancestry [1], [13]–[18]. These AIMs-based studies are complemented by reports of *continental* group ancestry across a variety of populations of the Americas using markers in uniparentally inherited mitochondrial DNA (mtDNA, for example [2], [3], [19]–[26]) and the non-recombining portion of the Y-chromosome (NRY, [27]–[29]) or a combination of both (for example [6], [30]–[35]). While these mtDNA and NRY markers are not suitable for assessing the ancestry of an individual, group ancestry based on the combination of mtDNA and NRY often correlates with the average AIMs-based ancestry [1], [5], [7]. Also, unlike AIMs, these markers are powerful tools for predicting maternal/paternal population demographic processes [36]–[38] and have comprehensive published resources covering all populated continents.

These studies of continental ancestry in the Americas conclude that individual admixture, and often group ancestry, varies extensively between geographically distinct groups united by the same ancestral label. The existing admixture frequently has a distinct gender bias, showing a larger contribution from European males and African/Native American females across multiple groups of the whole continent [5], [39], [40] (with the exception of European Americans [4], [7]). The Native American component that is generally small in North America [5] plays a significant role in Central and South America [39]. Focusing on African-derived populations, US African Americans were described to have a significant and variable proportion of individual European ancestry. While on average this European ancestry falls within the 15–25% range [1], [5], [15], regional differences were reported among some African American groups (the lowest level of European admixture (3.5%) was reported in Gullah Sea Islanders [41]). In Central and South America, in addition to varying European admixture, a variable Native American component adds to the ancestral complexity, making the populations of the Americas distinct from each other in their continental admixture.

Our interest lies in the African component of this continental admixture. The contribution of African ancestry to American populations was previously investigated using historical resources as well as genetic markers, mainly mtDNA. These reports suggest that there are ancestral contributions from 2–3 large African regions: West (W), West-Central/South-West (WC/SW) [3], and possibly South-East (SE) Africa [6] and their proportion differs between North, Central and South America. This implies that continental admixture is not the only source of genetic differences between geographically distinct populations in the Americas of African ancestry, but within-Africa admixture may play a significant role as well.

More recently, reports using autosomal markers and focusing on US African Americans have also been published. Bryc *et al*. investigated the ancestry of 365 US African Americans from across the United States and concluded that their ancestry is most similar to non-Bantu Niger Kordofanian-speaking populations of W/WC Africa based on analysis including 12 populations [40]. Zakharia *et al.* showed that the individual ancestry of 136 African Americans, investigated using 450,000 autosomal SNPs, is drawn mainly from West and West-Central Africa and, unlike the European component, this proportion is not very variable [42]. However, African variation was represented either by populations expected to contribute little to present-day US African Americans or by Yoruba, Mandenka, and Bantu – three populations representing the hundreds of populations of W/WC/SW Africa. While these AIMs-based studies have done a thorough analysis using current-day resources, they are limited by both their low within-Africa resolution that may reduce the complexity of the within-Africa component in African American ancestry as well as a narrow focus within the Americas.

While it has been previously reported that the contribution of W and WC/SW African populations varies between African-descended populations from North, Central and South America [2], [3], there remains limited information about the underlying reasons for these differences. To address this, we first comprehensively characterized African genetic diversity on the population level. Defining diverse African groups helped us to estimate with unprecedented resolution their contribution to admixed African-descended American populations of North and South America, and the Caribbean. By using a systematic approach to understand the source of African ancestry we have shown that genetically distinct African populations contributed differently to the genetic pool of geographically distinct American populations of African descent. Interpretation of our results suggests how this genetic ancestry-based pattern reflects the different colonial history of each region.

## Results

### Continental Ancestry of African Americans

#### mtDNA and NRY

**Figure 1:**
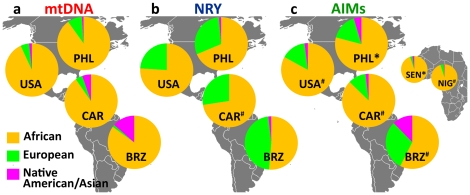
Pan-continental group ancestry of African-descended Americans. (a) mitochondrial DNA, (b) Y-chromosome (reflecting maternal and paternal admixture, respectively), (c) Ancestry Informative Markers (AIMs, reflecting the autosomal genome), showing the relative ratio of the three world populations that significantly contribute to the admixed populations of the United States (represented here as the entire US or by our sample from Philadelphia), the Caribbean Islands, and Brazil. The diverse socio-cultural histories of South and North America are reflected in sex-specific admixture and in overall admixture levels that differ between North and South America. The autosomal genome admixture proportions (c) approximately reflect the combination of the maternal and paternal contributions (a and b). AIMs-based estimates for Senegalese and Nigerian samples (far right) were added for comparison of African ancestral vs. American admixed populations. (Note: * designates samples typed and analyzed by the authors, while # designates previously published estimates. The remaining sample sets were collected from the literature as raw sequence and/or haplotype data and analyzed by the authors.)

Using comprehensive databases (**File S1** and **File** **S2**) assembled from published mtDNA and NRY marker data, we have calculated the continental group admixture in American populations of primarily African ancestry sampled from Philadelphia, across the United States, the Caribbean, and Brazil (see **Table S1** and **Table S2** in **File S3** for the list of populations and publications mined for mtDNA and NRY marker data, respectively). We confirmed that the previously described [4], [5], [7] European gender-specific admixture and a North-South gradient are present. European males, rather than females, are predominantly responsible for the European genomic contribution to American populations of African descent and both Native American females and European males provided a greater contribution to South American (represented by Brazil) compared to US admixed populations (**Figure 1** **,** and **Table S3** in **File S3**).

#### Autosomal Ancestry Informative Markers (AIMs)

To complement the group-specific information of uniparental (mtDNA and NRY) markers, we typed 175 autosomal AIMs (**File S4**) to estimate the continental individual admixture proportions in a larger set of Philadelphia African Americans (n = 331, **Figure 1**), Philadelphia European Americans (n = 728, **Figure S1** in **File S3**), and Africans from Senegal (n = 205, **Figure 1**) (these include all of the Philadelphia samples subjected to mtDNA and NRY analysis). Consistent with historical records, we see substantial inter-individual variability in admixture in the African Americans, with estimated African ancestry ranging from 7% to ∼100% (average 79.1%). In comparison, European ancestry in European Americans rarely dropped below 85%.

Further, we compared the AIMs-based group ancestry estimates (obtained by averaging the individual ancestry estimates) with the estimates calculated here based on published mtDNA and NRY variation in all African-descended American groups (see **Table S3** in **File S3**). We found that the African proportions of ancestry based on AIMs or averaged mtDNA and NRY estimates are correlated (**Figure 1**, and **Table S4** in **File S3**) with the exception of Brazil, possibly because diversely admixed Afro-Brazilian populations were sampled for each marker. These results demonstrate that sub-Saharan African ancestry can easily be separated from European and Native American-Asian ancestry and that ancestry estimates based on mtDNA/NRY and AIMs are highly correlated if the populations are thoroughly sampled.

### Within-Africa Ancestry: Maternal Contribution

#### MtDNA variation within Africa

**Figure 2:**
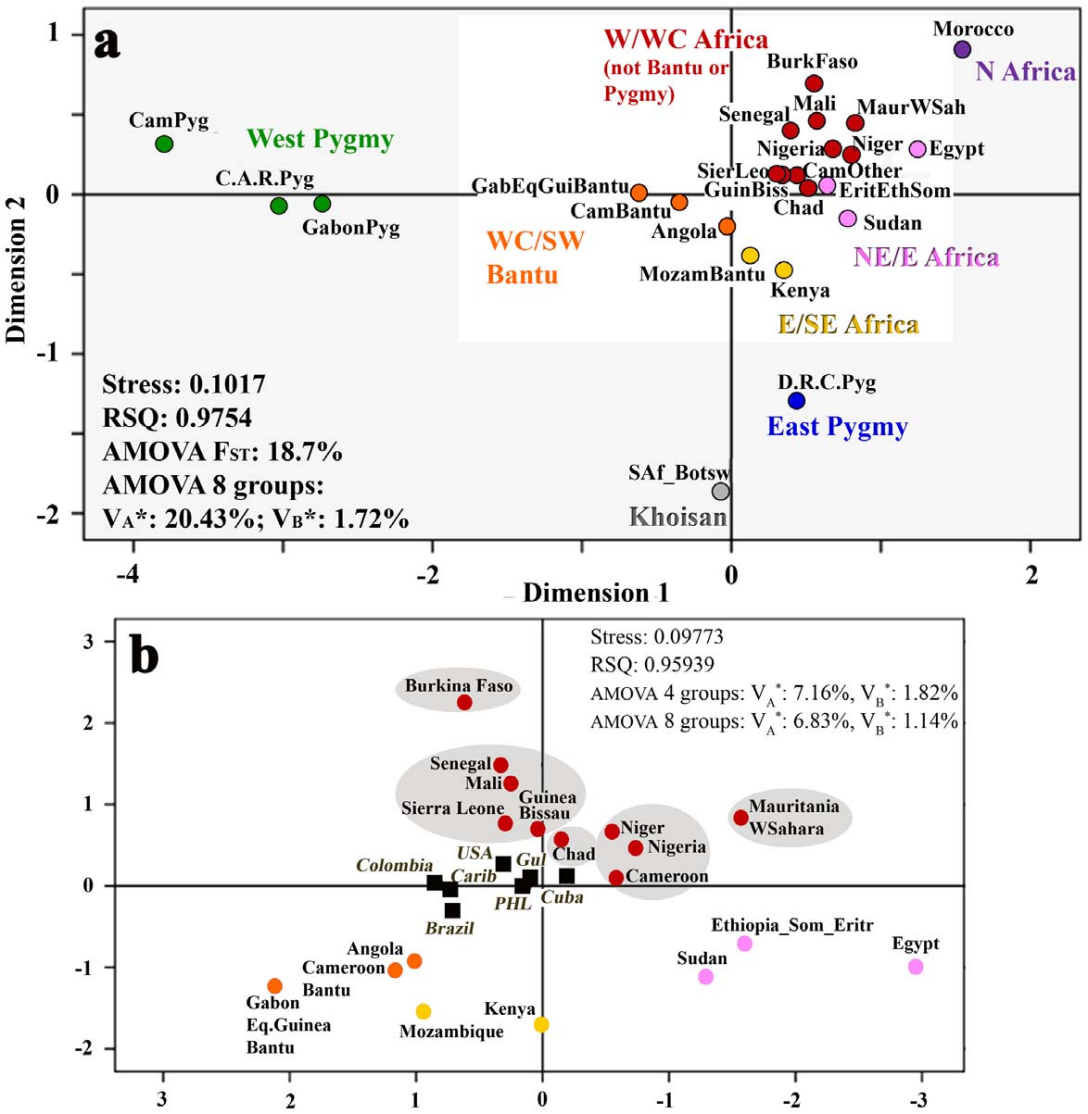
Multidimensional Scaling Plot of African mtDNA variation. (a) MDS plot of mtDNA variation within Africa. Africa was divided based on geography combined in a few cases with ethnicity (in the case of Pygmy hunter gatherers and Bantu speakers). SAMOVA was used to eliminate the following outlier groups: West Pygmy (Cameroon, C.A.R., and Gabon), Khoisan speakers (South Africa and Botswana), East Pygmy group Mbuti (D.R.C.), and Moroccan sample (mainly Berbers from North Africa). After removing outliers, the remaining states were divided using SAMOVA into 4 groups: WC/SW Bantu speakers from Angola, Cameroon, Gabon and Equatorial Guinea, E/SE sample from Kenya and Mozambique Bantu speakers, NE/E sample from Egypt, Sudan, Eritrea, Ethiopia, and Somalia, and W/WC countries after excluding Bantu speakers and Pygmy hunter-gatherers. The MDS plot parameters and AMOVA results are listed in the left bottom corner. When all 24 populations are considered, 18.7% of variation is between these 24 groups. When the populations are grouped into 8 groups, 20.43% of total variation is captured between these groups and 1.72% within these groups. (b) MDS plot showing the mtDNA variation-based genetic distances between African populations after the outliers (West and East Pygmy, Khoisan speakers and North Africans, outside of lighter insert in a) were excluded from the calculations. This plot shows the general structure of the remaining regions (with highlighted W/WC clustering) and their relationship to the admixed African American populations, depicted using only the African portion of their ancestry.

In order to relate within-Africa genetic variation to admixed Americans of African descent, we first assessed the genetic similarity of African populations using published African mtDNA variation. We initially divided the African continent into geographical regions, using current African countries as independent units except in the case of the populations of Cameroon, Democratic Republic of Congo (D.R.C.), Central African Republic (C.A.R.), and Gabon, where ethnic affiliation was also considered and these countries were further divided into Bantu, Pygmy, and “other” populations. We used SAMOVA [43] to first identify several genetically distinct groups (**Figure 2a**): a) West Pygmy from Cameroon, C.A.R. and Gabon, b) Khoisan speakers from South Africa and Botswana, c) Individuals from D.R.C. that consisted mainly of East Pygmy Mbuti, and d) Moroccans (mainly Berbers) from North Africa. After excluding the outliers from the calculations (West and East Pygmy, Khoisan speakers and North Africans, outside of lighter insert in **Figure 2a**), the remaining countries were split by SAMOVA into 4 groups (**Figure 2b**): 1) West Central/South West (WC/SW) Bantu from Angola, Cameroon, Gabon and Equatorial Guinea, 2) East/Southeast (E/SE) African individuals from Kenya and Mozambique Bantu speakers, 3) Northeast/East (NE/E) African individuals from Egypt, Sudan, Eritrea, Ethiopia, and Somalia, and finally 4) West/West Central (W/WC) countries after excluding Bantu speakers and Pygmy hunter-gatherers that cluster closely when divided by countries (details in **Table S5** in **File S3**). These relationships are parallel to the published genetic structure based on the autosomal polymorphic markers [44].

#### mtDNA variation in West/West-Central Africa

We were interested to investigate the W/WC African population in more depth. We have assembled a large amount of data that includes over fifty ethnic groups sampled from 9 W/WC countries, Chad (C) and Mauritania/Western Sahara (NW), yet this region seemed to be relatively homogeneous when dissecting mtDNA pool within the whole of Africa. Our goal was to define clusters within W/WC African populations composed of data from Burkina Faso, Cameroon, Chad, Guinea-Bissau, Mali, Mauritania, Niger, Nigeria, Senegal, Sierra Leone, and Western Sahara that would group genetically similar units based on the information in our database: language, geography or ethnic affiliation. We evaluated clustering using multi-dimensional scaling and AMOVA methods [43], maximizing the between-group variation (v_A_) and minimizing the within-group variation (v_B_).

First, five geographically defined clusters were identified: 1) Mauritania and Western Sahara, 2) Burkina Faso, 3) Niger, Nigeria, and Cameroon, 4) Guinea-Bissau, Mali, Senegal, and Sierra Leone, and 5) Chad (where v_A_ = 2.29%, v_B_ = 0.45%, **Figure 2b** and **Figure S2** in **File S3**). Second, three language-defined clusters were identified: 1) Mande and Atlantic North/South speakers of the Niger-Congo family, 2) Berber and Semitic speakers of the Afro-Asiatic family, and 3) heterogeneous cluster grouping speakers of Nilo-Saharan, Chadic of the Afro-Asiatic family and non-Bantu Volta-Congo of the Niger-Congo family (v_A_ = 2.26%, v_B_ = 0.31%, **Figure S3** in **File S3**). Finally, we grouped W/WC Africa by ethnicity (see **Figure S4**, **Figure S5**, and **Text S1** in **File S3**, and **File S5**). While we have evaluated the within-Africa data in a variety of ways, each grouping provides additional information while none proved to be superior. Therefore, we used all three clustering approaches (by geography, language, and ethnicity) in our admixture analysis but for simplicity, we refer mainly to clustering by geography in the main text.

### Within-Africa ancestry of admixed populations of African descent

We first established which of the previously identified eight African clusters depicted in **Figure 2a** contributed significantly to the admixed American populations using ADMIX software. Then, we dissected these regions further to obtain high within-Africa resolution when estimating the contribution of specific African regions to the admixed populations. We tested our approach on admixed populations from archipelagos off the African coast.

#### Admixed Populations in Africa

To confirm that we can correctly assess the African contribution to admixture in American populations, data from two geographical regions off the W/WC coast of Africa, Cabo Verde and São Tomé e Príncipe, were evaluated (**Figure S6** and **Table S6** in **File S3**). These two archipelagos were former Portuguese hubs of the Atlantic slave trade and historical records of contributing African populations are available [45]. Since these archipelagos have relatively small populations with well-described histories, they can serve as a kind of natural control analysis for subsequent analysis of the larger and more diverse populations of the Americas. Our admixture analysis indicated that the current population of Cabo Verde derives solely from West Africa (∼100% from W/WC, not including Bantu speakers or Pygmy), namely from West Niger-Congo speakers of Guinea-Bissau, Senegal, and Sierra Leone (∼90%) and Semitic/Berber speakers of Mauritania, Mali and Western Sahara (∼10%). In contrast, the founding population of São Tomé e Príncipe is drawn from both SW/WC Bantu (40–46%, mainly from Gabon/Equatorial Guinea and Angola) and West Africa (54–60%). The West African portion is drawn from the same populations as seen in Cabo Verde, or possibly from the population of Cabo Verde itself [45]. However, the available data do not cover the Ivory and Gold Coast that may be represented by this source. Our results, based on genetic variation, are consistent with the most likely source populations based on geographical proximity and historical records. The strong relationship between genetic variation, geography, and historical record supports the hypothesis that the admixture analysis used here is a reasonable approach for predicting within-Africa ancestry.

#### African-derived populations in the Americas

**Figure 3:**
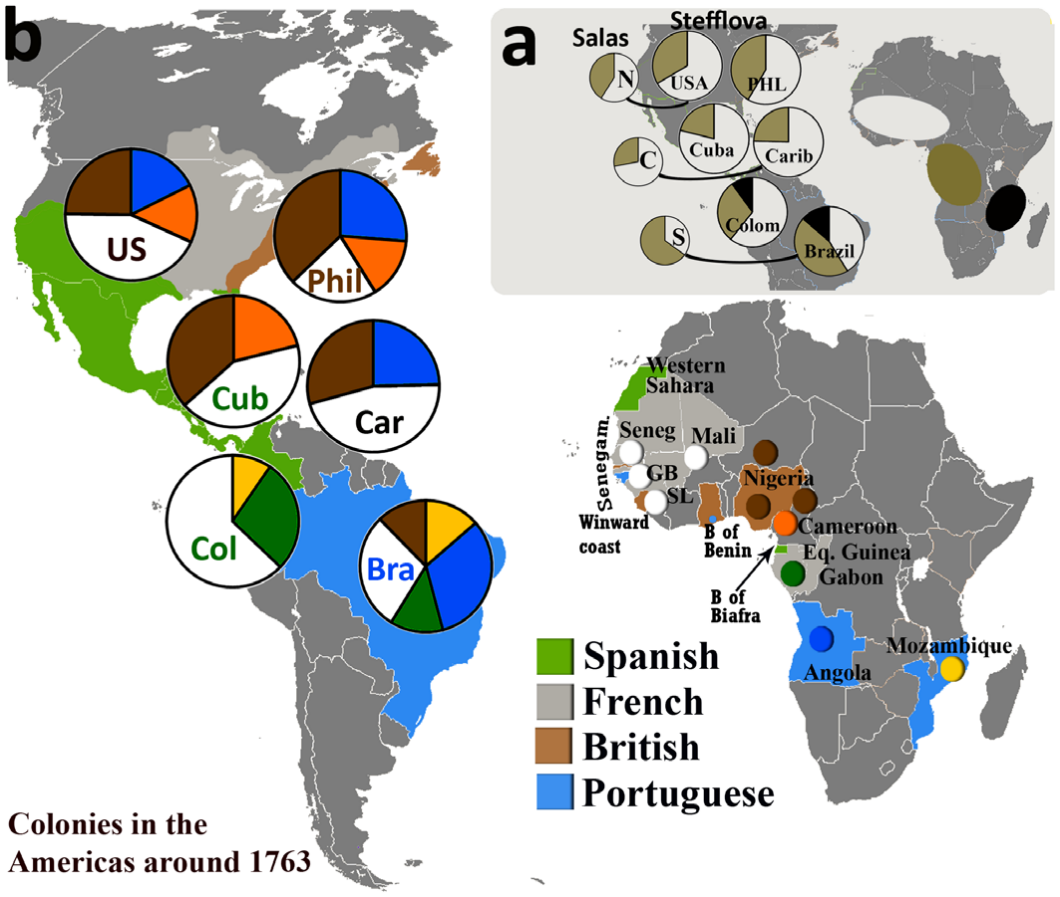
African regions contributing to the populations of the Americas. (a) Left: proportion of W/WC Africa contributing to the mtDNA pools of North (N, USA), Central (C, Caribbean), and South (S, Brazil) America as reported by Salas et al. [3]. Right: the proportion of W/WC, SW/WC Bantu and SE Africa contributing to mtDNAs in comparative populations of America analyzed in this paper (“Stefflova”). The relative contributions of these large African regions agree with Salas et al. [3], except for the Brazilian sample that displays significant input from SE Africa as well, which may have been included in WC portion by Salas et al. (b) Within-Africa mtDNA variation of admixed populations of Americas within the context of the colonial system. Maps are colored, depicting the relevant British (brown), French (light gray), Portuguese (blue), and Spanish (green) colonies in the Americas around 1763 and Africa and overlaid with pie charts depicting the relative proportions of mtDNA variation in the pool of African-derived mtDNAs of USA, Philadelphia, Caribbean, Cuba, Colombia, and Brazil derived from corresponding regions of W/WC, SW/WC Bantu, and SE Africa. Enrichment of American variation from source African colonies is particularly visible on Brazil and Philadelphia that have drawn most of their variation from Portuguese or British colonies. (*Note: GB = Guinea Bissau; SL = Sierra Leone; Seneg = Senegal, Senegam. = Senegambia; B = Bight, Eq. = Equatorial.)

Guided by admixture coefficients obtained from ADMIX, we found that only W/WC Africa, SW/WC Bantu, and SE Africa contributed significantly to the genetic ancestry of admixed Americans (**Figure 3a**). There is a varying ratio between contributions from W/WC Africa vs. SW/WC Bantu to the populations of America such that the contribution of W/WC Africa is the greatest in Cuba (79% vs. 21%) and the Caribbean (75% vs. 25%), less in the United States (68% vs. 32%) and Philadelphia (59% vs. 41%), and even less in Brazil (41% vs. 45%), although Colombia does not follow this C>N>S trend (63% vs. 28%). In addition, Brazil and Colombia show significant contribution from SE Africa (14% and 10%, respectively). We proceeded to investigate in greater depth which regions of Africa contribute to American admixture in order to explain this Central-North-South variation. We assessed the contribution of the geographically, linguistically, and ethnically defined groups within these large African regions to each admixed American population (**Table S6** in **File S3**).

**Figure 3b** depicts both the colonial powers in the Americas and Africa as well as the genetically defined regions within Africa that contributed to the pool of mtDNAs in the Americas (see **Table S7** in **File S3** for simplified relative contribution of African regions to the admixed populations of the Americas as represented in **Figure 3b**). When we traced the former colonies of Africa that contributed to genetic ancestry in former colonies in the Americas, we observed that the colonial systems and genetic marker data are related. A clear example is formerly Portuguese Brazil, where most of the regions contributing to the pool of African-Brazilians were drawn from former Portuguese colonies in Africa (see **Text S1** in **File S3** for summary of the historical context).

Because we are ultimately interested in capturing individual ancestry, we further investigated whether the diverse within-Africa ancestry can be captured by a set of AIMs suitable for estimating continental ancestry. We selected samples that had no more than 5% of European ancestry and used multidimensional scaling (MDS) analysis to evaluate differential clustering of West African Senegalese and Philadelphia African Americans, the latter having approximately 20% West African ancestry on average (**Figure S7** in **File S3**). As previously described when using a different set of European-African AIMs [46], we did not see any separation of these 2 clusters, suggesting that the within-Africa ancestry is not captured by markers which were selected for high informative value in predicting European-African ancestry. Therefore, while the currently used method of selecting AIMs is not designed to or capable of detecting the different African sources, mtDNA markers allowed us to identify which parental populations contributed heterogeneously to populations in the Americas.

## Discussion

The goal of this analysis was to investigate the differences in within-Africa genetic ancestry between the admixed groups of the Americas with African origin.

### Continental ancestry

We first considered how maternal and paternal continental ancestries that reflect gender-specific admixture patterns at the *group* level are correlated with the average *individual* ancestry represented by AIMs for each African-derived American population. Using mtDNA and NRY, we confirmed the presence of sex-specific admixture as well as the existence of differences across the Americas in continental admixture that are largely due to maternal contributions [4], [7]. So, while South American populations of both primarily African and European ancestry are highly admixed, in North America it is only the African American population that is highly admixed (**Figure 1**) compared to European Americans (**Figure S1** in **File S3**).

Based on AIMs and comparisons with other admixed populations, Philadelphia African Americans are, as expected, significantly more admixed than Senegalese or Nigerians [47] (average individual African ancestry in Philadelphia: 79.1%, Senegalese/Nigerians ∼95%), and the admixture profile resembles that of other African American groups in the US [47], [48] (79–83%). More importantly, we confirmed that the combined ancestry information is largely captured by uniparental markers and these can therefore not only provide insights into gender-specific admixture processes but also inform us about the source of the founding populations that contribute to admixture.

### Maternal ancestry within Africa

Africa is the most genetically diverse of the continents [49]. Since distinct groups of the Americas could have drawn the African portion of their ancestry from different populations within Africa, African “admixture” (in addition to *continental* admixture) can add to the diversity of these groups when population stratification is considered. In order to assess the within-Africa ancestry of African-derived Americans, possible source populations need to be defined from a thoroughly sampled genetic variation of Africa. We used mtDNA to evaluate the genomic variation contributed by populations from distinct African regions to American populations, since large source African mtDNA variation is already in place.

We assembled a comprehensive database of published mtDNA profiles, focusing on the African continent and admixed African-American populations. We have used this database to estimate the genetic ancestry and admixture proportions based on our well-defined map of correlation between geography-language-ethnicity and phylogenetically-relevant genetic distances. Our initial choice of separating Africa into geographically discrete regions was based on published work that reports correlation between genetic and geographic/linguistic distances in African populations [50]. Additionally, we separated Bantu and Pygmy, since the Bantu expansion was shown to weaken the language-genetic distance correlation [50]. Also, Pygmy and Bantu populations were shown to be distinct in their mtDNA signature [51], although Bantu males reduced this difference in NRY through an asymmetrical gene flow between Bantu males and Pygmy females [52].

### Within-Africa ancestry of populations of African descent

Our results allowed us to narrow down the founding groups that play a significant role in the within-Africa ancestry of African Americans. These groups are ancestrally found in the W, WC, SW, and SE regions of Africa, with the WC group split between Bantu and non-Bantu populations. There is a decreasing contribution from W/WC Africa in the order of C>N>S America such that the contribution of W/WC Africa is the greatest in the Caribbean (∼75%) and Cuba (∼79%), less in the United States (∼68%) and Philadelphia (∼59%), and even less in Brazil (∼41%) (the exception being Colombia with ∼63%). This is in agreement with published literature (Salas et al., [3]) that investigated the populations of the United States, the Caribbean, and Brazil and defined the African groups as W (our W and WC) and WC (our SW/WC Bantu and São Tomé e Príncipe), using 27 African haplotypes (see comparison in **Figure 3a**). Additionally, we observed a significant contribution from SE Africa to the African mtDNA pool of Brazil and Colombia (14% and 10%, respectively), perhaps because of greater phylogenetic resolution of our study (we defined 429 haplotypes). While the contribution of SE Africans to the Brazilian and Colombian pools was not reported in [3], it is corroborated by previous reports on Brazilians from São Paulo (∼12%) [6] and historical resources.

By undertaking a detailed phylogenetic analysis, we were able to *further* separate the contribution by various African regions into 7-10 genetically diverse groups/regions and estimate the proportions by which these regions contributed to the admixed African and American populations. We paid particular attention to the W/WC African variation, since West Africa was historically considered to be a highly significant source of slaves to North America. First, we compared the African profiles of the admixed populations of archipelagos off the W/WC Africa coast (Cabo Verde and São Tomé e Príncipe), followed by the Americas (USA, Philadelphia, Cuba, Caribbean islands, Colombia, and Brazil). The population of Cabo Verde is mainly drawn from the Senegambia/Guinea/Windward coast directly neighboring the archipelago. In São Tomé e Príncipe, ∼40% of mtDNAs were drawn from nearby Gabon and Equatorial Guinea and Angola, and ∼60% from the West coast region (or possibly Cabo Verde). Both of these regions were major sources of slaves for Portuguese colonies at the time of populating this archipelago (**Figure S6** in **File S3**), connecting it to the parental African regions not only by a geographical distance but also by the Portuguese control of both the source and target regions.

### Colonial systems and genetic ancestry

Detailed analysis of the populations of the New World revealed a marked difference in the source of African mtDNAs between North and South America, Caribbean, and neighboring regions.

Closer examination of these within-Africa ancestry estimates reinforced the strong relationship between the colonial systems of Africa and the Americas and present-day genomic ancestry. The Portuguese were the pioneers of the slave trade and the main importers of African slaves into Brazil. The Portuguese started bringing slaves into Brazil by the end of the 16^th^ century, mainly from the Upper Guinea and Kongo-Angola regions. But, the majority (∼80%) of the slaves was brought during the 18^th^ and 19^th^ centuries, where Guinea Bissau and Angola were the major sources of slaves. Towards the end of the slave trade, Mozambique contributed significantly as well as Bight of Benin (mainly US import). The current genetic variation of Brazilian populations reflects these geographical and historical sources: Angola and Gabon/Equatorial Guinea (32% and 13%, respectively) represent the majority of Brazilian ancestry, followed by the Senegambia/Guinea-Bissau/Sierra Leone region (29%), SE Africa (13.6%), and Nigeria, Niger, Cameroon (12.3%) (**Figure 3b**). In concordance with these genomic estimates, assessment from the historical record suggests these regions contributed ∼50-60% (Angola/Gabon/Equatorial Guinea), 20–25% (Upper Guinea, comprising mainly Guinea Bissau to Sierra Leone), 10–15% (SE Africa) and 10-18% (Bight of Benin) [45].

While the Portuguese had colonies both in Africa and the Americas, Spain lacked the same presence, holding a virtual monopoly in the Americas and almost no colonies in Africa. As a result, we expected to observe significant heterogeneity in African ancestral sources between Spanish colonies in the Americas – mainly between the mainland and islands, populated in different time periods [45]. For example, Colombia carries the signature of both the very early (Guinea-Bissau (63%), Kongo-Angola (27%, represented by Gabon/Equatorial Guinea)) and late (SE Africa (10%)) Portuguese/Spanish slave sources. In contrast, the majority of slaves were brought to Cuba at the end of the 18^th^ and beginning of the 19^th^ centuries. These individuals originated primarily from the Bight of Benin, Biafra, and Western Guinea [45] (see **Figure S6** in **File S3** for map), represented by genomic ancestry from Nigeria/Niger/Cameroon (37%), Cameroon Bantu (21%), and Guinea Bissau/Senegal/Sierra Leone/Mali (42%), respectively.

A different distribution of African ancestry was observed in Philadelphia, a former British colony. The ancestry of African Americans from Philadelphia draws its mtDNAs mainly from the Bight of Biafra and Benin regions (37% Nigeria-Niger-Cameroon and 15% Cameroon Bantu in Philadelphia compared to 25% and 14% in the US overall, respectively). Ancestry from Guinea Bissau-Mali-Senegal-Sierra Leone predominates in other United States African American populations compared to Philadelphia alone (43% vs. 22%). Despite the differences in coverage and sampling, this pattern may be attributed to a significant contribution of slaves from British colonies in Africa to the British-controlled Philadelphia region compared to a more diverse contribution to other parts of the United States from French, Spanish, and Dutch colonies. Additional possible contributing factors include the different periods of the slave trade influencing the Philadelphian population compared to the other parts of the United States. However, these remain tentative conclusions since we cannot rule out a contribution from sampling bias. Another example of these differences is the Gullah/Geechee populations from South Carolina/Georgia that have >78% of their source from the Guinea Bissau-Mali-Senegal-Sierra Leone region (data not shown), corresponding to the “Rice coast” around Sierra Leone that was the major source of slaves drawn by the United States in the later period of the slave trade [21], [45].

Our data also included evaluation of the Caribbean islands of Dominica, Grenada, St. Kitts, St. Lucia, St. Vincent, and Trinidad. The majority of slaves were brought to these islands during the boom of sugar trading at the end of the 18^th^ and beginning of the 19^th^ centuries. The observed mtDNA variation reflects the multiple colonial powers that controlled these islands, with possible unique composition of within-Africa ancestry for each island.

### Limitations of our study

Our database and analyses have several limitations. First, there remains limited data from W/WC Africa, where the published literature does not cover Ivory and Gold Coasts. Thus, the analysis of genotype data is limited by the available published data. Also, our data suggest that genetic variation captured by the mtDNA genotypes (HVS I/II and part of the coding region) may not, despite the effort invested in defining a large set of haplotypes, contain sufficient information to accurately separate many genetically similar ethnic groups, especially those within West Africa. Second, mtDNA is a single locus that can inform us only about group maternal ancestry and needs to be complemented with study of NRY and AIMs. While NRY analysis is complicated by limited resolution and coverage of the published data in Africa as well as Bantu speakers' migrations [50], additional detailed AIMs studies are on their way to help inform these analyses [40], [42], [44], especially once a more thorough coverage of African variation is in place.

### Conclusion

We have dissected the ancestry of African-descended Americans at the level of continental and within-Africa ancestry. Our detailed analysis of the African mtDNA landscape helped us, for the first time, to identify the maternal ancestry of African-descended populations to the several (6–7) regions *within* W/WC, SW/WC and SE Africa. We estimated the contribution of each of these African regions to the American populations and linked this variation with historical records. Our results suggest that the distribution and identity of within-Africa ancestral contributions to groups of African descent in the Americas correspond to colonial histories and slave trade routes. The present analysis of genetic variation implies that African populations contributed differently to distinct populations of the New World, suggesting that the assumption of genetic homogeneity of African ancestry within the Americas is not necessarily valid. In addition, the selection of ancestral markers, including AIMs selected to account for continental or European vs. African admixture only, may not be adequate to detect or control for the heterogeneity in African source populations. This has significance for epidemiology studies using self-identified race as a proxy for ancestry in association studies, since this term does not capture the genetic admixture both on the continental level (as shown previously) but also on the within-Africa level.

## Materials and Methods

### Database

We have collected marker data to evaluate the continental (i.e., European, Asian, Native American, and African) and within-African ancestry. We have assembled extensive databases of published mitochondrial DNA (mtDNA) and non-recombining Y chromosome (NRY) genotype and haplotype information from world populations linked to geography, language (obtained from http://www.ethnologue.com/) and ethnicity information. This database includes ∼13,800 mtDNA sequences (**File S1**) and ∼9,050 NRY haplogroup affiliations (**File S2**) with a strong focus on including comprehensive African data. We have also included admixed populations of the Americas, mainly those that were self-identified as having primarily African ancestry (here designated as African-descended Americans) but also some mixed or predominantly white populations.

The mtDNA database is a comprehensive compilation of the relevant literature that could be used for a deep phylogenetic analysis. For a list of publications and detailed breakdown of African and American populations included in the mtDNA database see **File S3** (**Table S1**, **Text S1** and **References S1**).

For the NRY database, we assembled multiple sample sets typed for NRY single nucleotide polymorphisms (SNPs), focusing mainly on those publications that genotyped the phylogenetic relationships with similar or greater depth as in our dataset (see **Table S2** in **File S3** for the list of publications included). We limited our consideration of these datasets because shallower NRY typing significantly reduces the resolution that could be achieved using the combined dataset.

In all ancestry estimates, we used the phylogenetic relationship between haplotype data thoroughly characterized for both mtDNA and NRY. Specifically for mtDNA, 429 pan-continental mtDNA haplotype motifs were defined based on the variation within the mtDNA database. Of these, approximately 5,800 African individuals (including admixed individuals from Cabo Verde and São Tomé e Príncipe) from 10 geographic regions, 13 language families, 33 countries, and 143 populations were used to capture 304 haplotypes that represent variation within Africa. These data were used to map the group ancestry of African Americans to smaller regions of Africa.

### Population comparisons

Arlequin 3.11 [53] was used to estimate genetic distances utilizing the phylogenetic relationship defined by 429 FASTA-formatted mtDNA haplotypes, assuming Tamura and Nei's [54] model for nucleotide substitution. Analysis of molecular variance (AMOVA) [55] was used to assess the between group and within-population variation for each step. SAMOVA 1.0 software [43], combining AMOVA with geographical information, was used to explore the clustering of geographic regions or ethnic groups of the whole and W/WC Africa based on the genetic variation.

### Autosomal AIMs

We typed 175 AIMs (**File S4**) for 331 self-identified African Americans and 728 European Americans from Philadelphia, and 205 Senegalese using an Illumina Golden Gate Platform. The individuals from Philadelphia were ascertained between 1995 and 2007 as part of a prostate cancer case-control study, with cases identified through Urologic Oncology Clinics at multiple hospitals of the University of Pennsylvania Health System (UPHS) and controls being men attending UPHS general medicine clinics. The individuals from Senegal were identified and ascertained from university and hospital populations in Dakar, Senegal. All study subjects from US and Senegal provided written informed consent for participation in this research. IRB approval for this study has been provided by the Committee on Studies Involving Human Beings of the University of Pennsylvania (Protocol #3614-2) and by the Commission Ethique et Evaluation at the Hopital General de Grand Yoff in Dakar (FWA 00002772).

The primary set of AIMs consisted of 149 SNPs that were selected from Tian et al. [16] to address the European admixture by maximizing Fishers Information Coefficients (FIC) based on three admixture scenarios [56] (i.e., 10%/90%, 50%/50%, and 90%/10% European/African contribution). We also typed two additional AIMs sets based on the published sets from Lao et al. [17] (9 SNPs) and Reiner et al. [15] (17 SNPs). The Lao additional panel allowed us to further explore Native American-SE Asian ancestry. The individual level ancestry was estimated using STRUCTURE [57], [58] with 10,000 burn-in cycles and 50,000 replicates under the admixture model for 3 populations (see LnP(D) for K = 1–5 in **Table S8** in **File S3**) and including control “parental” individuals of known African, European (selected individuals with <2% admixture from the Senegalese and European American pool) and Asian ancestry (Native American and Asian populations are related more closely and for K = 3, we use Asian ancestry as a surrogate for Native American ancestry). To calculate Native American-SE Asian ancestry, we have included individuals of known Asian (n = 33) or admixed ancestry (n = 10, mainly European-Asian) as additional controls. We obtained the group ancestry simply by averaging the individual ancestry estimates for each group.

### MDS plots

Multidimensional scaling (MDS) plots were constructed using SPSS with input data in the form of an Arlequin-generated matrix of Slatkin's linearized F_ST_ distances [59], incorporating the phylogenetic relationship among the 429 mtDNA haplotypes. For each MDS plot, we report the stress and RSQ statistics, which summarize the goodness of fit of multidimensional data in 2 dimensions. Additionally, AMOVA was reported for the parental populations (indicated in each MDS figure) showing the percentage of variation captured by defining the language/geography/ethnicity groups. For **Figure S7** in **File S3**, the coordinates for MDS plot capturing Senegalese and US African Americans with <5% of European ancestry based on 175 AIMs were calculated using PLINK and plotted using Excel.

### Admixture estimates

The group level admixture based on uniparental markers was estimated using ADMIX 2.0 [60], which incorporates both molecular divergence and haplotype frequencies. Both mtDNA and NRY were treated as a single locus. After 50,000/100,000 (mtDNA/NRY) bootstrap simulations, the data were reported as a percent contribution from a particular parental population along with an estimate of the sampling error (SD). Additional information about the groups that were chosen as parental populations in ADMIX-based admixture coefficient calculations is listed in **Text S1** in **File S3**. For continental admixture, we used complete profiles of admixed populations. For within-Africa admixture, we considered only the African-derived haplogroups (L, U6, U5b1b). For the admixed populations considered here, only 3 regions were shown to contribute: W/WC non-Bantu/non-Pygmy, Bantu of SW/WC, and SE Africa. These regions were further subdivided based on geography (SW/WC Bantu, **Figure 2**) or, in the case of W/WC, based on geography (**Figure S2** in **File S3**), language (**Figure S3** in **File S3**), and ethnicity (**Figure S4** and **Figure S5** in **File S3**).

## Supporting Information

File S1 mtDNA sequence information.
(2.90 MB XLS)

File S2 NRY marker information.
(0.11 MB XLS)

File S3 This file contains Tables S1–S8, Figures S1–S7, Text S1, and References S1.
(2.96 MB PDF)

File S4 AIMs.
(0.04 MB XLS)

File S5 W/WC ethnic groups with language affiliations.
(0.92 MB XLS)
