# Supplementary material for: Dissecting the Within-Africa Ancestry of Populations of African Descent in the Americas
Source: PLoS One. 2011 Jan 6;6(1):e14495. doi: 10.1371/journal.pone.0014495 (PMC3017210; doi:10.1371/journal.pone.0014495)
Supplement: File S3 — This file contains Tables S1–S8, Figures S1–S7, Text S1, and References S1. (2.96 MB PDF) [file pone.0014495.s003.pdf]

Table S1: List of publications and populations included into the database of mtDNA HVS I/II sequences.

Table S2: List of publications mined for NRY information.

Figure S1: Continental ancestry of European Americans and white Brazilians.

Table S3: Summary of estimates of continental ancestry presented in Figure 1 and Figure S1.

Table S4: Reported/Estimated vs. Calculated World admixture proportions.

Table S5: Geographically sorted African portion of mtDNA database.

Figure S2: MDS clustering of W/WC Africa divided by geography.

Figure S3: MDS clustering of W/WC Africa divided by languages.

Figure S4: MDS clustering of W/WC Africa divided by major ethnic groups.

Figure S5: MDS clustering of W/WC Africa divided by groups based on ethnical and language group affiliation.

Figure S6: Map of Africa with source W/WC, SW/WC Bantu, and SE African populations and their contribution to the admixed populations of São Tomé e Príncipe and Cabo Verde.

Table S6: The relative contribution of African regions to the admixed populations of the Americas and Africa.

Table S7: Relative contribution of African regions to the admixed populations as represented in Figure 3b.

Figure S7: MDS plot of Senegalese and Philadelphia African Americans based on AIMs.

Table S8: LnP(D) for STRUCTURE admixture estimate runs assuming 1-5 populations (K=1-5).

Text S1: Detailed description of mtDNA database, grouping of African ethnic groups, the historical context for African/American populations, and admixture analysis using ADMIX.

References S1

Table S1: List of publications and populations included into the database of mtDNA HVS I/II sequences.

Source	Continent	Region	Country	Group	Admixed	n	Relevance
Allard 2005 [1]	**Americas**	N	USA	African Americans	Admixed	765	Forensic database of African Americans in USA
Alves-Silva 2000 [2]	**Americas**	S	Brazil	Population of Brazil	Admixed	247	South American admixed population (mainly white)
Benn Torres 2007 [3]	**Americas**	C	Caribbean	African-Caribbeans	Admixed	313	Comparing African-descended Americans (1 sample omitted)
Ely 2006 [4]	**America**	N	USA	Gullah/Geechee	Admixed	78	Comparing African-descended Americans
Gonçalves 2008 [5]	**Americas**	S	Brazil	Black Brazilians (São Paulo)	Admixed	120	Comparing African-descended Americans
Guerreiro Jr. 2009 [6]	**America**	S	Brazil	White Brazilians	Admixed	171	Comparing European-descended Americans
Hűnemeier 2007 [7] Guerreiro Jr. 2009 [6]	**Americas**	S	Brazil	Black Brazilians (Porto Alegre)	Admixed	107	Comparing African-descended Americans (Hűnemeier reports African, Guerreiro adds European/Amerindian Hgs)
Hűnemeier 2007 [7]	**Americas**	S	Brazil	Black Brazilians (Rio de Janeiro)	Admixed	84	African (L) Hgs only
Mendizabal 2008 [8]	**Americas**	C	Cuba	Population of Cuba	Admixed	245	Central American admixed population
Salas 2008 [9]	**Americas**	S	Colombia	Afro-Colombians	Admixed	95	Comparing African-descended Americans
Sans 2006 [10]	**Americas**	S	Uruguay	Population of Uruguay	Admixed	43	General population of Uruguay
Silva 2006 [11]	**Americas**	S	Brazil	Black Brazilians	Admixed	50	Comparing African-descended Americans
Stefflova 2009 [12]	**Americas**	N	USA	African Americans	Admixed	217	Philadelphia African Americans
Stefflova 2009 [12]	**Americas**	N	USA	European-Americans	Admixed	204	Philadelphia European-Americans
Beleza 2005 [13]	**Africa**	SW	Angola	Bantu speakers	Native	110	People of Cabinda, Bantu expansion
Brandstätter 2004 [14]	**Africa**	E	Kenya	not reported	Native	93	Population of Nairobi
Brehm 2002 [15]	**Africa**	W	Cabo Verde	Population of Cabo Verde	Admixed	291	Displaced from Africa by Portuguese
Černý 2004 [16]	**Africa**	WC	N Cameroon	Chadic speakers	Native	103	Chadic speakers of E origin in WC Africa
Černý 2006 [17]	**Africa**	W	Burkina Faso	Fulbe	Native	97	W African Fulbe
Černý 2006 [17]	**Africa**	C/WC	N Cameroon, S Chad	Fulbe	Native	87	C Africa Fulbe
Černý 2007 [18]	**Africa**	WC	Cameroon	Fali, Kotoko, Masa	Native	79	Chadic and non-Bantu WC
Černý 2007 [18]	**Africa**	C	Chad	Arabs and Kanenbu	Native	77	Semitic and Nilo-Sah. WC
Černý 2007 [18]	**Africa**	WC	Niger	Buduma	Native	30	Chadic WC
Černý 2007 [18]	**Africa**	WC	Nigeria	Kanuri and Arabs	Native	69	Nilo-Saharan, Semitic WC

Chen 2000 [19]	Africa	S	South Africa	Khoisan speakers	Native	74	Khoisan hunter gatherers
Coia 2005 [20] (includes Destro-Bisol 2004 samples)	Africa	WC	Cameroon	Population of Cameroon	Native	440	Bantu and non-Bantu population of S and N Cameroon
Destro-Bisol 2004 [21]	Africa	C	CAR	Mbenzele Pygmies	Native	57	C African Hunter-gatherers
Ely 2006 [4]	Africa	W	Mali	Bambara and Malinke	Native	80	Bambara and Malinke ethnic groups of Mali
Gonzáles 2006 [22]	Africa	W	Mali	Multiple	Native	124	Variation in Mali pop.
Gonzáles 2006 [22] (includes Rando 1998 samples)	Africa	NW	Mauritania	Semitic speakers	Native	64	Semitic speakers of NW
Jackson 2005 [23]	Africa	W	Sierra Leone	Loko, Limba, Temne, Mende	Native	277	W Africa ethnic groups
Kivisild 2004 [24]	Africa	E	Ethiopia, Eritrea	Variation in E Africa	Native	270	E Africa Afro-Asiatic speakers
Mateu 1997 [25]	Africa	WC	Equat. Guinea	Bubi	Native	45	WC coast island inhabited by pre-colonial Africans
Mateu 1997 [25]	Africa	WC	São Tomé e Príncipe	-	Admixed	50	Displaced from Africa by Portuguese
Pereira 2001 [26]	Africa	SE	Mozambique	Bantu speakers	Native	109	Bantu-speaking SE Afr.
Plaza 2004 [27]	Africa	SW	Angola	Mbundu	Native	44	Source of Brazilian AAs
Quintana-Murci 2008 [28]	Africa	WC/C	Cameroon, Gabon, CAR	Babongo, Baka, Bakola, Bakoya, Biaka, Tigar	Native	382	West Pygmies
Quintana-Murci 2008 [28]	Africa	C	DRC	Mbuti	Native	39	East Pygmies
Quintana-Murci 2008 [28]	Africa	WC	Gabon, Cameroon	Bantu speakers	Native	983	Bantu speakers of WC Africa
Rando 1998 [29]	Africa	N	Morocco	Berber	Native	92	N Africans, mainly Berber
Rando 1998 [29]	Africa	NW	West Sahara	Semitic speakers	Native	25	NW Africans
Rando 1998 [29]	Africa	W	Senegal	multiple	Native	121	Population of Senegal
Richards 2000 [30]	Africa	NE	Egypt, Sudan	Egyptian, Nubian	Native	147	NE Africans
Rosa 2004 [31]	Africa	W	Guinea-Bissau	multiple	Native	372	Population of Guinea-Bissau
Salas 2002 [32]	Africa	SE	Mozambique	Bantu speakers	Native	308	Population of SE Africa
Silva 2006 [11]	Africa	C/WC	DRC, Cameroon	Bantu speakers	Native	20	WC and C Africa Bantu speakers
Stevanovitch 2004 [33]	Africa	NE	Egypt	Egyptian	Native	58	NE Africans
Stefflova 2009 [12]	Africa	W	Senegal	multiple	Native	49	Population of Senegal
Trovoada 2004 [34]	Africa	WC	São Tomé e Príncipe	Angolares, Forros, Tongas	Admixed	103	Displaced from Africa by Portuguese
Watson 1997 [35] (from Vigilant 1991)	Africa	S	Botswana	!Kung	Native	19	Khoisan speakers
Watson 1997 [35] (from Watson 1996)	Africa	E	Somalia, Kenya	Kikuyu, Somali, Turkana	Native	88	E Africans

Watson 1997 [35] (from Vigilant 1991)	**Africa**	C	CAR, DRC	Mbuti, Biaka Pygmies	Native	30	West and East African Pygmies
Watson 1997 [35] (from Vigilant 1991 and Watson 1996)	**Africa**	WC	Niger, Nigeria	multiple	Native	160	W and WC Africans
Watson 1997 [35] (from Graven 1995)	**Africa**	W	Senegal	Mandenka	Native	110	W African Mandenka
Kivisild 2004 [24]	**Arabian Peninsula**	-	Kuwait	Yemeni	Native	115	Transition between Africa and Eurasia
Richards 2000 [30]	**Arabian Peninsula**	-	multiple	Semitic speakers	Native	418	Transition between Africa and Eurasia
Fagundes 2008 [36]	**Americas**	S & N	multiple	Native Americans	Native	57	Native Americans
Li 2007 [37]	**Asia**	SE	China, Vietnam	Asians	Native	889	Asians
Malyarchuk 2003 [38]	**Europe**	SE	States of former Yugoslavia	Europeans	Native	248	Europeans
Richards 2000 [30]	**Europe**	All	Multiple	Europeans	Native	2802	Europeans
Richards 2000 [30]	**Eurasia/ Asia**	W	Iran, Armenia, N Caucasus, Azerbaijan, Turkey	-	Native	730	Eurasia

Table S2: **List of publications mined for NRY information.**

Source	Continent	Region	Country	Group	Admixed	n	Relevance
Brion 2005 [39]	**Americas**	S	Colombia, Argentina	mixed	Admixed	141	Admixed population of South America
Gonçalves 2008 [5]	**Americas**	S	Brazil	AA	Admixed	120	Admixed population of Brazil
Guerreiro Jr. 2009 [6]	**Americas**	S	Brazil	AA, EA	Admixed	257	Population of Brazil
Hammer 2006 [40]	**Americas**	N	USA	AA, NA, HA, EA, SA*	Admixed	2517	All US admixed populations
Hűnemeier 2007 [7]	**Americas**	S	Brazil	Black Brazilians (Rio de Janeiro)	Admixed	187	African Brazilians with partial information of European variation
Mendizabal 2008 [8]	**Americas**	C	Cuba	mixed	Admixed	132	Admixed population of Central America
Silva 2006 [41]	**Americas**	S	Brazil	mixed	Admixed	127	Population of Brazil
Stefflova 2009 [12]	**Americas**	N	USA (Philadelphia)	African Americans, European-Americans	Admixed	389	Admixed populations of USA
Vallone 2004 [42]	**Americas**	N	USA	African Americans, European-Americans	Admixed	229	Admixed populations of USA
Gonçalves 2005 [43]	**Islands**	NW	Acores	mixed	Admixed	121	Admixed population off the coast of Africa
Gonçalves 2005 [43]	**Islands**	NW	Madeira	mixed	Admixed	129	Admixed population off the coast of Africa
Gonçalves 2007 [44]	**Islands**	WC	São Tomé e Príncipe	multiple	Admixed	150	Admixed populations of Africa
Beleza 2005 [13]	**Africa**	SW	Angola/Cabinda	mixed	Native	74	Variation of Cabinda
Brion 2005 [39]	**Africa**	E/SE	Somalia, Mozambique	mixed	Native	235	Variation of E/SE Africa
Cruciani 2002 [45]	**Africa**	WC	Cameroon	multiple	Native	241	Variation of Cameroon
Cruciani 2002 [45]	**Africa**	C	C.A.R., D.R.C.	Biaka, Mbuti, Lissongo	Native	36	Pygmy populations
Cruciani 2002 [45]	**Africa**	W	Burkina Faso	Fulbe, Mossi, Rimaibe	Native	106	Variation of Burkina Faso
Cruciani 2002 [45]	**Africa**	S	South Africa	!Kung, Khwe	Native	90	Khoisan speakers
Cruciani 2002 [45]	**Africa**	N, E	Morocco, Ethiopia	Arabs, Berbers, Jews	Native	135	Semitic speakers
Luis 2004 [46]	**Africa**	N	Egypt	Arabs	Native	147	Variation of Egypt
Luis 2004 [46]	**Africa**	E	Rwanda, Kenya, Tanzania	Bantu, Hutu, Tutsi, Wairak	Native	235	Variation of East Africa
Luis 2004 [46]	**Africa**	WC/W	Cameroon, Benin	Bamileke, Bantu, Fon	Native	199	WC/W Africa
Robino 2007 [47]	**Africa**	N	Algeria	Arabs	Native	102	Variation of North Africa

Rosa 2007 [48]	**Africa**	W	Guinea-Bissau	Niger-Congo speakers	Native	282	West African variation
Tishkoff 2007 [49]	**Africa**	E/WC	Tanzania, Nigeria	Hadza, Sandawe, Yoruba, Turu, Mbugwe, Datog, Burunge	Native	232	Variation of Tanzania
Wood 2005 [50]	**Africa**	E	Ethiopia, Uganda, Tanzania, Kenya	Amhara, Ganda, Kikuyu, Luo, Maasai, Oromo, etc	Native	159	Variation of E Africa
Wood 2005 [50]	**Africa**	C	D.R.C., C.A.R.	Alur,Baka, Biaka, Hema, Mbuti, Nande	Native	141	Variation of C Africa
Wood 2005 [50]	**Africa**	S	South Africa, Namibia, Zimbabwe	!Kung, Ambo, Dama, Herero, Nama, Shona, Sotho, Tsumkwe, Zhosa, Zulu	Native	322	Variation of S Africa
Wood 2005 [50]	**Africa**	WC	Cameroon	multiple	Native	161	Variation of Cameroon
Wood 2005 [50]	**Africa**	W	Mali, Ghana, Senegal/Gambia	Dogon, Ewe, Fante, Ga, Mandenka, Wolof	Native	219	Variation of W Africa
Wood 2005 [50]	**Africa**	N	Egypt, Tunisia	mixed	Native	120	Variation of N Africa
Rosa 2007 [48]	**Africa**	W	Guinea-Bissau	Balanta, Bijagos, Felupe, Fulbe, Mandenka, Nalu, Papel	Native	282	Variation of Guinea Bissau
Luis 2004 [46]	**Arabian Peninsula**		Oman	Arabs	Native	121	En route out of Africa
Brion 2005 [39]	**Europe**	W/SW	Germany, Denmark, Galicia	mixed	Native	430	Variation of W Europe
Gonçalves 2005 [43]	**Europe**	SW	Portugal	mixed	Native	303	Variation of SW Europe
Brion 2005 [39]	**Eurasia**	W	Turkey	mixed	Native	51	
Brion 2005 [39]	**Asia**	SE/E	China, Japan, Thailand	mixed	Native	179	Variation of SE/E Asia
Brion 2005 [39]	**Greenland**			mixed		90	

*AA=African Americans, EA=European-Americans, NA=Native-Americans, HA=Hispanic-Americans, SA=Asian-Americans

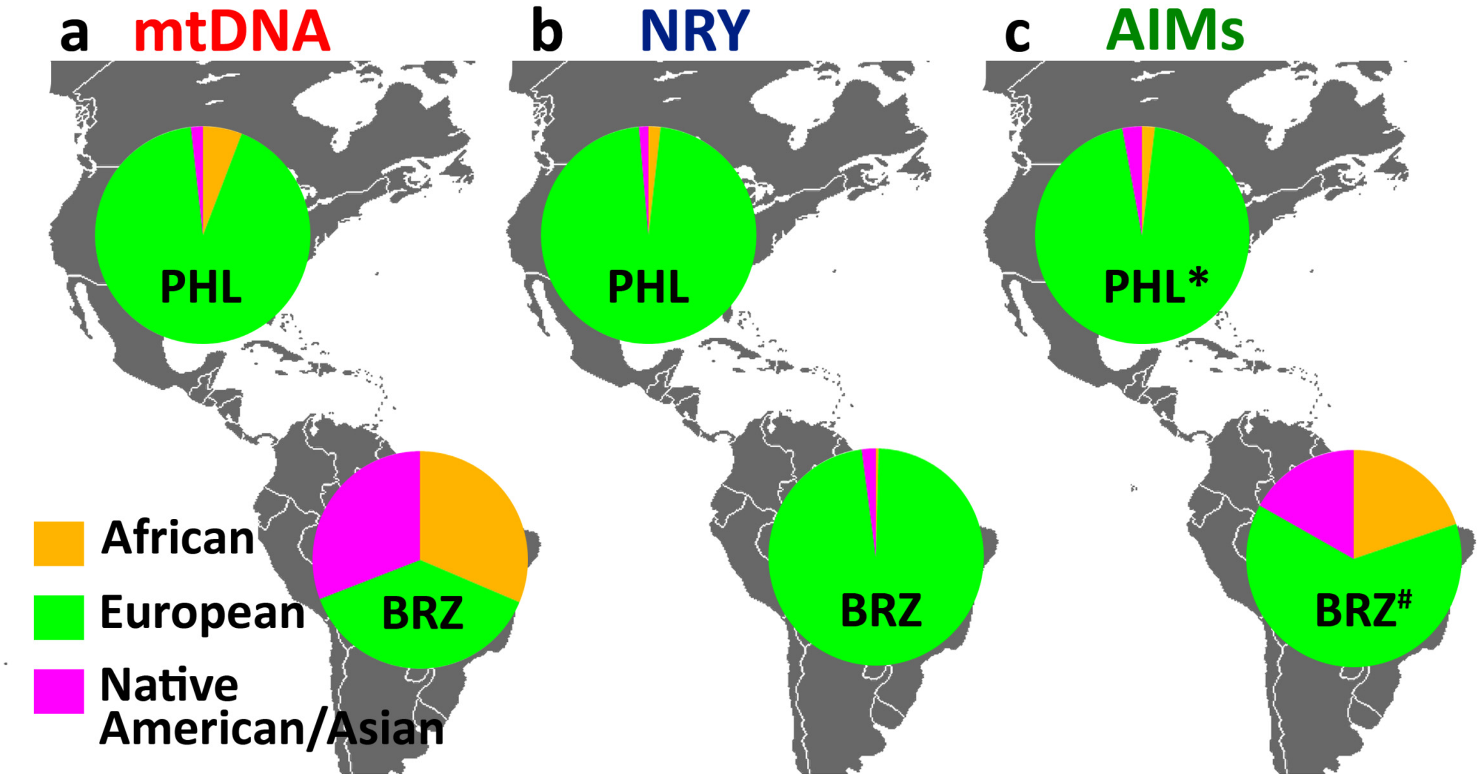

Figure S1: Continental ancestry of European Americans and white Brazilians estimated using: **a)** mitochondrial DNA, **b)** Y-chromosome, (reflecting maternal and paternal admixture, respectively), **c)** Ancestry Informative Markers (reflecting the autosomal genome) showing the relative ratio of the three world populations that significantly contributed to the admixed populations of Philadelphia and Brazil. This figure complements **Figure 1**, showing significantly higher admixture in South America that is strongly sex-biased. (Note: * designates samples that were typed and analyzed by authors, # designates previously published estimates and the remaining sample sets (mtDNA and NRY of European Americans from Philadelphia and white Brazilians) were collected from literature as a raw data (see comprehensive databases in **File S1** and **File S2**) and analyzed by authors).

Table S3: **Summary of estimates of continental ancestry presented in Figure 1 and Figure S1** assessed by: 1) typing and analyzing using STRUCTURE program ("type+structure") or 2) literature mining of the mtDNA-, NRY-, and AIMs-based estimates ("published") or 3) assembling the raw mtDNA/NRY data that were subjected to admixture analysis ("admix") using our comprehensive database for defining the parental populations (if multiple references were used, samples were first pooled and then analyzed as a single group). Values are reported as relative contribution from African, W Eurasian (mtDNA/NRY) or European (AIMs) and Native American (mtDNA/NRY) or Native American/SE Asian populations (AIMs*, except in [51] reporting Native American ancestry only) ± SE (which is defined as sampling error for "admix", standard error for "published" (if reported) and pseudo-standard error for "type+structure" where pSE=[1/2 (Σ PI)]/1.645). For those cases where we estimated mtDNA/NRY-based admixture using "admix", we had to define the ancestral African, European and Native American/Asian parental populations. The details of which populations were included in the mtDNA analysis can be found in **Text S1**. For NRY, simply all populations from **Table S2** with designation in column "Continent": *European/African/Asian* and "Admixed": *Native* were used. The details of "type+structure" methods, including the description of parental populations, is listed in Methods section under "Autosomal AIMs".

Region	Markers	N	Americans of African descent			N	Americans of European descent			Lit. (analysis or published estimates)
			African	European	Nat.Am./As.		African	European	Nat.Am./As.	
Brazil	mtDNA	277	85%± 3.4	*1.4% ± 1*	13.6% ± 3.2	247	30.7%± 6	38.4% ± 5	30.9% ± 4.6	[5,7,11]/ [2] (admix)
	NRY	182	51.3% ± 4	46.8% ± 4	*1.8% ± 1.9*	203	*0.4% ± 1*	97.4% ± 2	*2.1% ± 1.8*	[5,6]/ [6] (admix)
	AIMs	499	57.6%	30.4%	12.4%	308	19.8%	63.5%	16.8%	[53,6] (published)
Caribbean	mtDNA	313	90.3% ± 2.4	4.3% ± 1.8	5.3% ± 1.7	-	-	-	-	[3] (admix)
	NRY	354	73%	27%	-	-	-	-	-	[3] (published)
	AIMs	298	87.3% ± 2.4	10.8% ± 2.6	*1.9% ± 2.4*	-	-	-	-	[52] (published)
Philadelphia	mtDNA	217	90.1%± 2.9	8.8% ± 2.8	*1.1% ± 0.8*	204	*5.8%± 5*	92.4% ± 7	*1.8%± 1*	[12] (admix)
	NRY	199	67.5% ± 4	31.2% ± 4	*1.3% ± 1.5*	190	*1.8% ± 1*	96.7% ± 2	*1.4% ± 1*	[12] (admix)
	AIMs*	331	79.1% ± 2.8	16.3% ± 3.1	4.6% ± 3.4	728	*1.9%±1.5*	95.2%±3.3	*2.9% ± 3.2*	(type+structure)
USA	mtDNA	765	93.1%± 1.2	5.2% ± 1.4	*1.7% ± 0.6*	-	-	-	-	[1] (admix)
	NRY	653	76.5% ± 2	24.5% ± 2	0	-	-	-	-	[40] (admix)
	AIMs	50	83.0% ± 1.6	14.7% ± 1.8	2.3% ± 0.5	-	-	-	-	[51] (published)
			Africans							
Senegal	AIMs*	205	94.9% ± 1.7	*2.9% ± 1.5*	*2.2% ± 2*	-	-	-	-	(type+structure)
Nigeria	AIMs	40	95.1% ± 1.1	3.65% ± 1.1	1.25% ± 0.4	-	-	-	-	[51] (published)

Table S4: Reported/Estimated vs. Calculated World admixture proportions. Average group admixture proportions estimated using ancestry informative markers (AIMs) for US populations (Yeager 2008 [51]), Caribbean Islands (Ben-Torres 2008 [52], using WLS method (upper) and ML method (lower)), Brazil (Salzano & Bortolini 2002 [53]) and Philadelphia (Stefflova 2009 [12]) compared to the "calculated" values, obtained by averaging the mtDNA and NRY contribution (m_{AIMs}= ½ m_{mtDNA} + ½ m_{NRY}) from **Table S3**. Despite the reported and calculated values are very similar, Native American/Asian contribution is over-estimated for populations with < 5% of the "true" contribution. This is most likely due to two factors: 1) uncertainty in predicting low levels of admixture from population that has a significant overlap of variation in chosen AIMs with the European population, 2) difference in definition of source populations, where mtDNA and NRY estimates separate the Native American and group Europe and West Asia whereas our AIMs-based estimates group SE Asians with Native Americans. Also, the reported admixture proportions for Brazilian populations are slightly different from the calculated values, probably due to low number of typed AIMs as well as sampling of diversely admixed population for mtDNA and NRY vs. AIMs reports.

		Reported			Calculated from mtDNA+NRY		
Descent	Geography	Africa	Europe	N.Amer/As	Africa	W Eurasia	N.Amer
primarily African	USA	83.0%	14.7%	2.3%	84.5%	14.7%	0.8%
	Philadelphia	79.1%	16.3%	4.6%	78.8%	20%	1.2%
	Caribbean	82.3%	12.7%	5%	81.7%	15.7%	2.6%
		87.3%	10.8%	1.9%			
	Brazil	57.6%	30.4%	12.4%	67.4%	24.7%	7.9%
primarily European	Philadelphia	1.9%	95.2%	2.9%	3.8%	94.6%	1.6%
	Brazil	19.8%	63.5%	16.8%	15.6%	67.9%	16.5%

Table S5: Geographically sorted African portion of mtDNA database.

Group	Geo. region	Ethnic group (n)	Country	Language	n	Source
West Pygmy	WC	Baka (127), Bakola (88), Tigar (35)	Cameroon	Bantu and non-Bantu Volta-Congo	250	Quintana-Murci 2008 [28]
	WC	Babongo (45), Bakoya (31)	Gabon	?	76	Quintana-Murci 2008 [28]
	C	Biaka (73), Mbenzele (57)	C.A.R.	Bantu	130	Quintana-Murci 2008 [28], Watson 1997 [35], Destro-Bisol 2004° [21]
Khoisan	S	!Kung (62), Kwe (31)	Botswana, South Africa	Khoisan	93	Chen 2000 [19], Watson 1997 [35]
East Pygmy	C	Mbuti (52)	D.R.C	Nilo-Saharan	52	Quintana-Murci 2008 [28], Watson 1997 [35]
N Africa	NW	Berber (60), other (32)	Morocco	Afro-Asiatic	92	Rando 1998 [29]
E/SE Africa	SE	Bantu speakers	Mozambique*	Bantu	417	Pereira 2001 [26], Salas 2002 [32]
	E	Turkana (37), Kikuyu (24), other (93)	Kenya	Nilo-Saharan, Bantu, ?	154	Brandstätter 2004 [14], Watson 1997 [35]
NE/E Africa	E	Tigrai (45 and 8)	Ethiopia, Eritrea	Semitic**	53	Kivisild 2004 [24]
	E	Somali (27)	Somalia	Cushistic**	27	Watson 1997 [35]
	E	Amhara (120), other (97)	Ethiopia	Afro-Asiatic	217	Kivisild 2004 [24]
	NE	Egyptian	Egypt	Afro-Asiatic	125	Stevanovitch 2003 [33], Richards 2000 [30]
	NE	Nubian	Sudan	Nilo-Saharan	80	Richards 2000 [30]
WC/SW Bantu	SW	Mbundu (44), other (110)	Angola-Cabinda	Bantu	154	Beleza 2005 [13], Plaza 2004 [27]
	WC	Bakaka (50), Bamileke (48), Bassa (46)	Cameroon	Bantu	144	Coia 2005 [20]
	WC	Ewondo (78)	Cameroon	Bantu	78	Quintana-Murci 2008 [28], Coia 2005 [20]
	WC	Fang (39), Ngumba (88), other (10)	Cameroon	Bantu	137	Quintana-Murci 2008 [28], Silva 2006 [11]
	WC	multiple (17 groups)	Gabon	Bantu	831	Quintana-Murci 2008 [28]
	WC	Bubi of Bioko island	Equator. Guinea	Bantu	45	Mateu 1997 [25]
	C	Bantu speakers	D.R.C.	Bantu	10	Silva 2006 [11]
	W/W C/C	***Fulbe, Peul, Poular, Tukulor***	Burkina Faso, Nigeria, Cameroon N, Guinea-Bissau, Mali, Senegal, Chad	Atlantic N	402	Černý 2006 [17], Coia 2005 [20], Gonzales 2006 [22], Rando 1998 [29], Rosa 2004 [31], Stefflova 2009 [12], Watson 1997 [35]
	W	***Balanta***	Guinea Bissau	Atlantic N	63	Rosa 2004 [31]
	WC/C	***Arabs*** (Chad, Shuwa)	Nigeria, Chad	Semitic	65	Černý 2007 [18]

**W/WC Africa (not Bantu or Pygmy) ‡**	W	***Bambara***	Mali, Senegal	Mande	79	Gonzales 2006 [22], Ely 2006 [4], Rando 1998 [29], Stefflova 2009 [12]
	WC	***Fali***	N Cameroon	non-Bantu Volta	81	Černý 2007 [18], Coia 2005 [20]
	C	***Kanembu***	NW Chad	Nilo-Saharan	50	Černý 2007 [18]
	WC	***Kanuri*** Δ	Niger, Nigeria	Nilo-Saharan	45	Černý 2007 [18], Watson 1997 [35]
	W	***Limba***	Sierra Leone	Atlantic S	68	Jackson 2005 [23]
	W	***Malinke***	Mali, Senegal	Mande	93	Ely 2006 [4], Gonzales 2006 [22], Rando 1998 [29]
	W	***Mandenka***	Guinea-Bissau	Mande	142	Rosa 2004 [31], Stefflova 2009 [12], Watson 1997 [35]
	W	***Mende***	Sierra Leone	Mande	57	Jackson 2005 [23]
	W	***Temne***	Sierra Leone	Atlantic S	122	Jackson 2005 [23]
	W	***Wolof***	Senegal	Atlantic N	59	Rando 1998 [29], Stefflova 2009 [12]
	W	multiple†	***Guinea-Bissau, Senegal***	***Atlantic N***	247	Rando 1998 [29], Rosa 2004 [31], Stefflova 2009 [12]
	WC	Hide (23), Kotoko (56), Mafa (31), Masa (32)	***Cameroon (East origin)***	***Chadic***	142	Černý 2004 [16], Černý 2007 [18]
	WC	Daba (20), Mandara (37), Podokwo (39),Uldeme (27)	***Cameroon (other)***	***Chadic***	123	Coia 2005 [20]
	WC	Buduma (30), Hausa (20)	***Niger***	***Chadic***	50	Černý 2007 [18], Watson 1997 [35]
	W	Soninke (11), Jancanca (1), Bobo (5), Loko (30), Sussu (8), Soce (1), 4 unknown	***Guinea-Bissau, Mali Senegal, Sierra Leone,***	***Mande***	60	Gonzales 2006 [22], Jackson 2005 [23], Rando 1998 [29], Rosa 2004 [31]
	W/WC	Dogon (6), Ivorienne (1), Senoufo (1), Tali (20), Tupuri (25), Yoruba (31)	***Cameroon, Nigeria, Mali, Senegal***	***non-Bantu Volta-Congo***	86	Coia 2005 [20], Gonzales 2006 [22], this paper, Watson 1997 [35]
	NW/WC/W	Maure (2), Tuareg (25), W Saharan (25), unknown (65)	***Mauritania, Niger, W Sahara, Senegal***	***Semitic/Berber***	116	Gonzales 2006 [22], Rando 1998 [29], this paper, Watson 1997 [35]

*Samples from Salas 2002 are mainly from Mozambique but also include samples from bordering neighbors, ** Semitic, Cushistic, Berberic and Chadic language groups all belong to Afro-Asiatic macro-language family, *** Bold-italic font indicates a group designation, Δ Kanuri were combined with overlapping samples from Cameroon with East origin, † Bainouk (1), Baiote (6), Banhu (1), Beafada (19), Bijagò (22), Brame (8), Cassanga (6), Diola (29), Landoma (1), Lebou (4), Manjaco (32), Mankanya (20), Mansonca (18), Nalu (26), Papel (23), Sahalle (1), Serer (30), ‡ Songhai/Sonrhai - Nilo-Saharan speakers of Nigeria and Mali (10+6) – were left out of the analysis

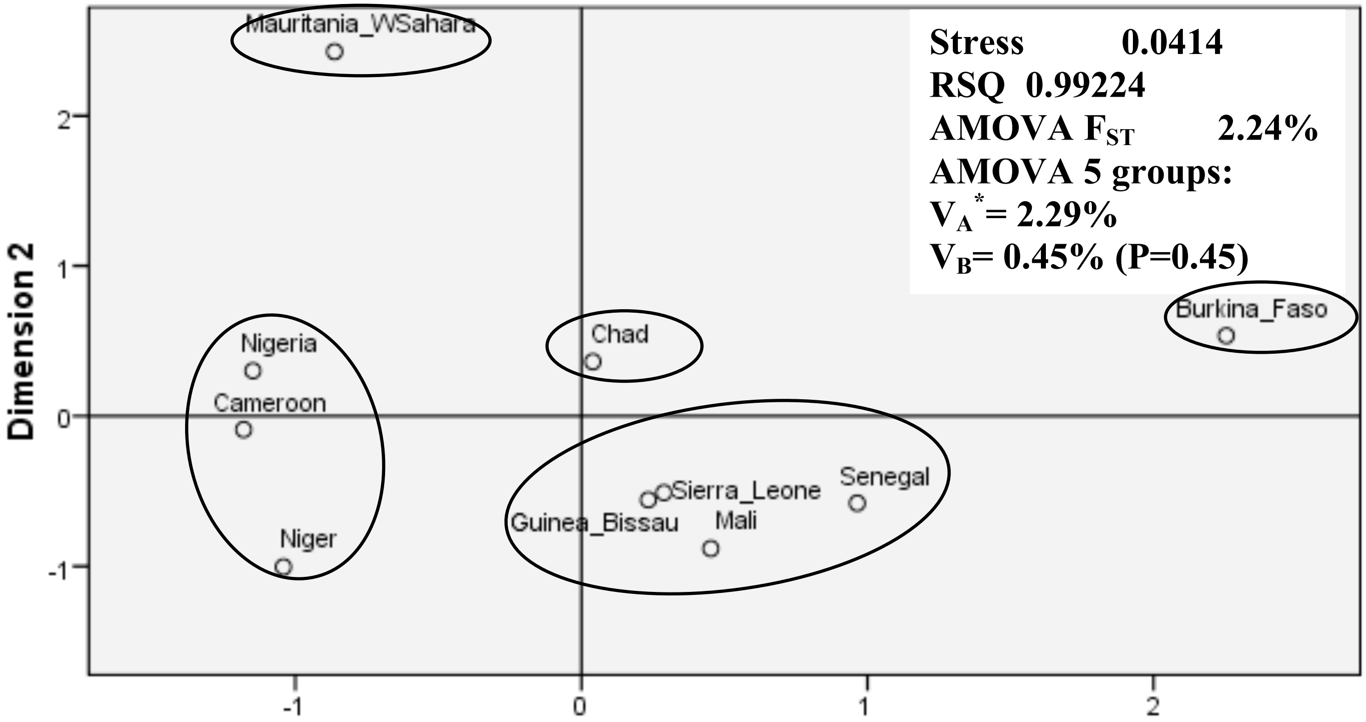

Figure S2: **MDS clustering of W/WC Africa divided by geography.** We first divided our mtDNA dataset of non-Bantu speaking and non-Pygmy populations of NW/W/WC/C Africa based on geographical location/political clusters into 10 countries: Burkina Faso, Cameroon “other”, Chad, Guinea-Bissau, Mali, Mauritania + Western Sahara, Niger, Nigeria, Senegal, and Sierra Leone. Guided by F_{ST} P values and SAMOVA, the mtDNA variation in these 10 states can be grouped into 5 clusters: 1) NW African Mauritania/Western Sahara, 2) Burkina Faso, 3) Nigeria clustered with Cameroon “other” and Niger, 4) W African Guinea-Bissau, Mali, Senegal, and Sierra Leone cluster, with 5) Chad being potentially grouped with these countries or the 5th group (with significant “among group variation” V_A contributing to 2.29% of total variation, and “among populations within groups” V_B contributing to 0.45% of total variation with p=0.45).

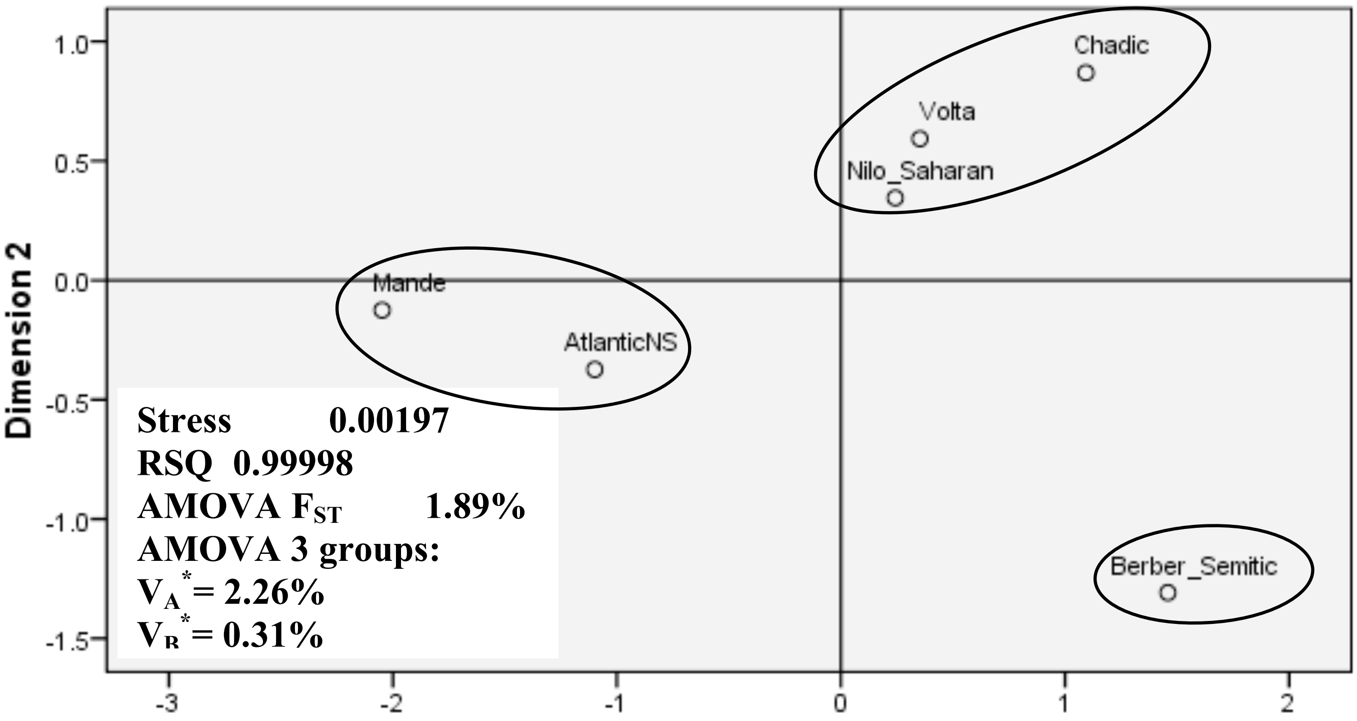

Figure S3: **MDS clustering of W/WC Africa divided by languages.** We next divided our mtDNA dataset of non-Bantu speaking and non-Pygmy populations of NW/W/WC/C Africa into 6 groups based on language affiliation: Afro-Asiatic speakers of Chadic (Cameroon, Niger) and Berber/Semitic (grouped because of low numbers and similarity; mainly from Mauritania and Nigeria) language groups, Nilo-Saharan speakers (mainly Chad and Nigeria), and Niger-Congo speakers of Atlantic South and North (majority belong to Fulbe), Mande and non-Bantoid Volta-Congo (mainly of Cameroon and Nigeria) sub-language groups. The mtDNA variation in these 6 sub-language groups can be grouped into 3 clusters: 1) Chadic + Nilo-Saharan + non-Bantoid Volta-Congo speakers, 2) Mande + Atlantic North and South, 3) Berber and Semitic speakers (with "among group variation" V_A contributing to 2.3% of total variation, and "among populations within groups" V_B contributing to 0.31% (P=0.01) of total variation, both P<0.05).

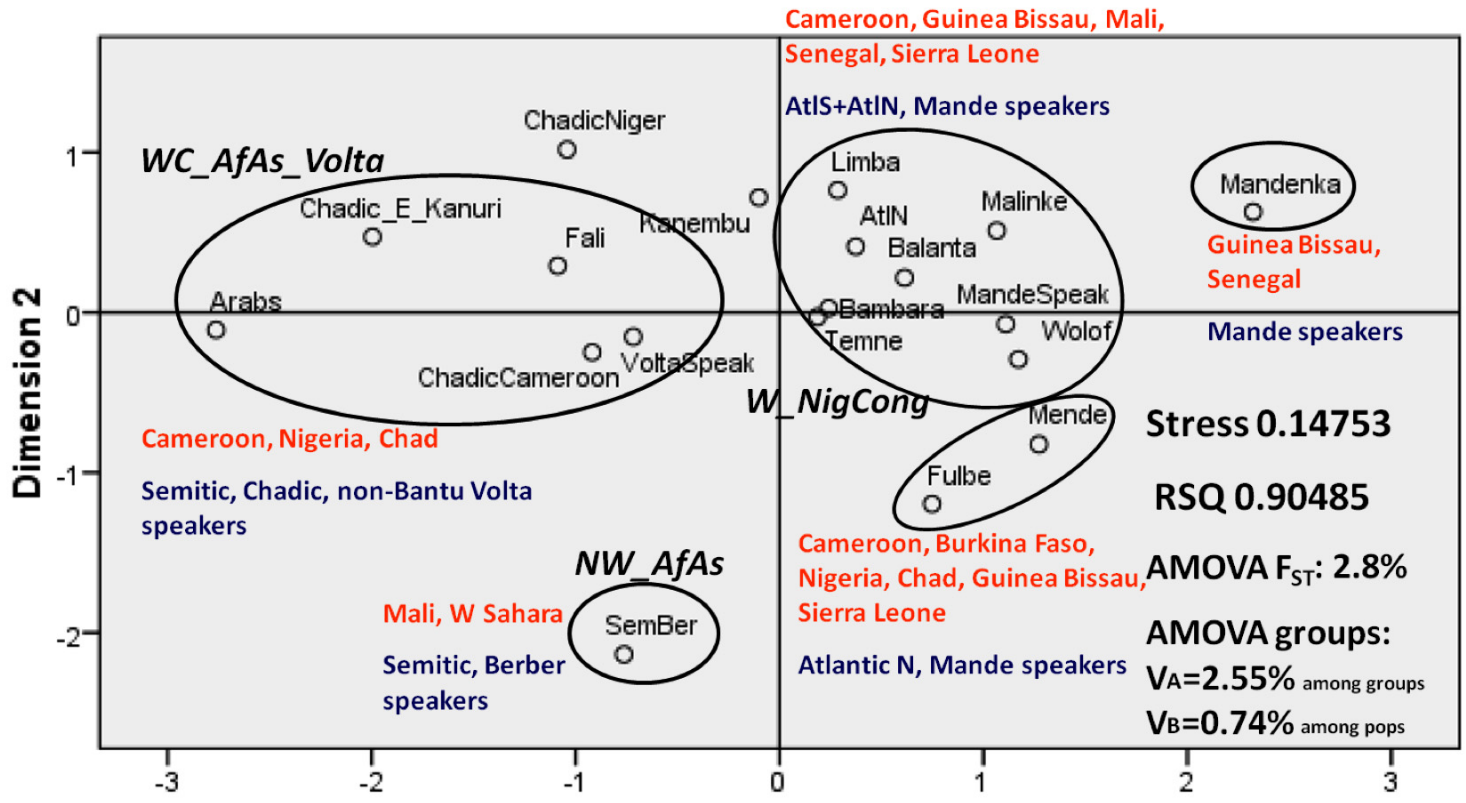

Figure S4: **MDS clustering of W/WC Africa divided by major ethnic groups.** We separated the mtDNA dataset of non-Bantu speaking and non-Pygmy populations of NW/W/WC/C Africa into 19 groups based on ethnicity as follows: single ethnic groups with sufficient (n ≥ 50) sample size: **Balanta** (n=63), **Bambara** (n=79), **Fulbe** (n=402), **Fali** (n=81), **Kanembu** (n=50), **Limba** (n=68), **Malinke** (n=93), **Mandenka** (n=142), **Mende** (n=57), **Temne** (n=122), **Wolof** (n=59); followed by groups comprising different ethnicities with common language/geography/origin: **AtlN** (multiple ethnic groups of Guinea-Bissau and Senegal, speaking Atlantic North, n=247), **Chadic_E_Kanuri** (Chadic speakers from Cameroon of East origin: Hide (n=23), Kotoko (n=56), Mafa (n=31), Masa (n=32), and Kanuri (n=45)), **ChadicCameroon** (other Chadic speakers from Cameroon: Daba (n=20), Mandara (n=37), Podokwo (n=39),Uldeme (n=27)), **ChadicNiger** (Chadic speakers from Niger: Buduma (n=30), Hausa (n=20)), **MandeSpeak** (Mande

Figure S4 legend (continued)

speakers of Guinea-Bissau, Senegal, and Sierra Leone: Soninke (n=11), Jancanca (n=1), Bobo (n=5), Loko (n=30), Sussu (n=8), Soce (n=1), 4 unknown), **VoltaSpeak** (non-Bantoid Volta-Congo speakers of Cameroon, Nigeria, Mali, and Senegal: Dogon (n=6), Ivorienne (n=1), Senoufo (n=1), Tali (n=20), Tupuri (n=25), Yoruba (n=31)), and **SemBer** (Semitic and Berber speakers of Mauritania, Niger, West Sahara, Senegal, Niger: Maure (n=2), Tuareg (n=25), W Saharan (n=25), unknown (n=65)). The mtDNA variation in these 19 groups can be grouped into 5 clusters, representing the WC Afro-Asiatic and Volta-Congo speakers, NW Afro-Asiatic speakers, W Niger-Congo speakers, Fulbe+Mende and Mandenka, excluding ChadicNiger and Kanembu for insufficient sample size to stand alone (with "among group variation" V_A contributing to 2.55% of total variation, and "among populations within groups" V_B 0.74%, both $P<0.05$).

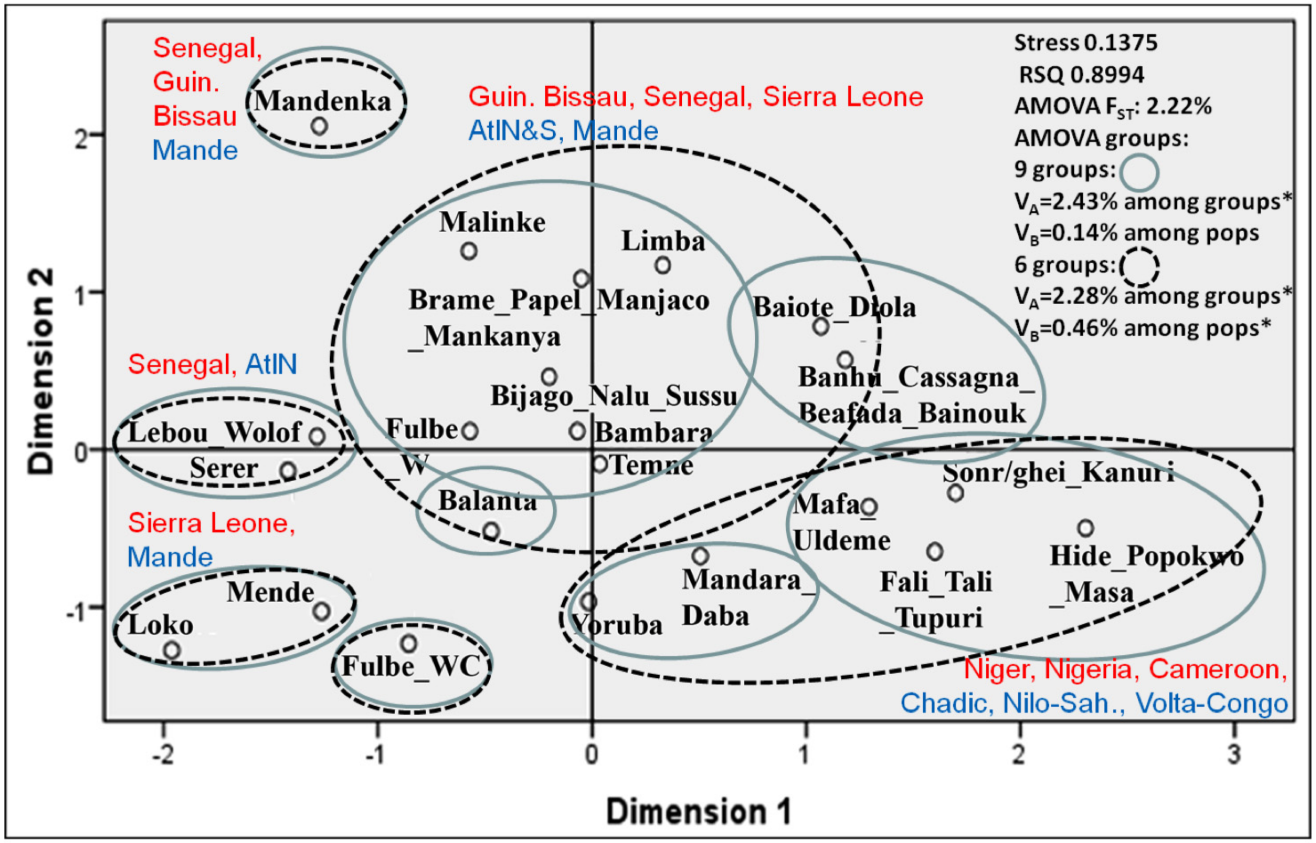

Figure S5: **MDS clustering of W/WC Africa divided by groups based on ethnical and language group affiliation.** Finally, we explored a new subdivision of W/WC/NW/C cluster based on ethnicity combined with language group info and partial geography, divided into 22 groups (as listed in **File S5**). These groups can be ideally clustered into 9 groups (the variation between the ethnic groups of each macro-group V_B is not significant), but Yoruba+Mandara_Daba (n=82), Balanta (n=63), and Baiote_Diola+Banhu_Cassagna_Beafada_Bainouk (n=62) have small number of samples to stand alone in admixture analysis and therefore 6 groups were used for estimating the admixture coefficients (based on SAMOVA grouping). The MDS plot characteristics and AMOVA either for all samples or 9 or 6 clusters are listed in the top right corner.

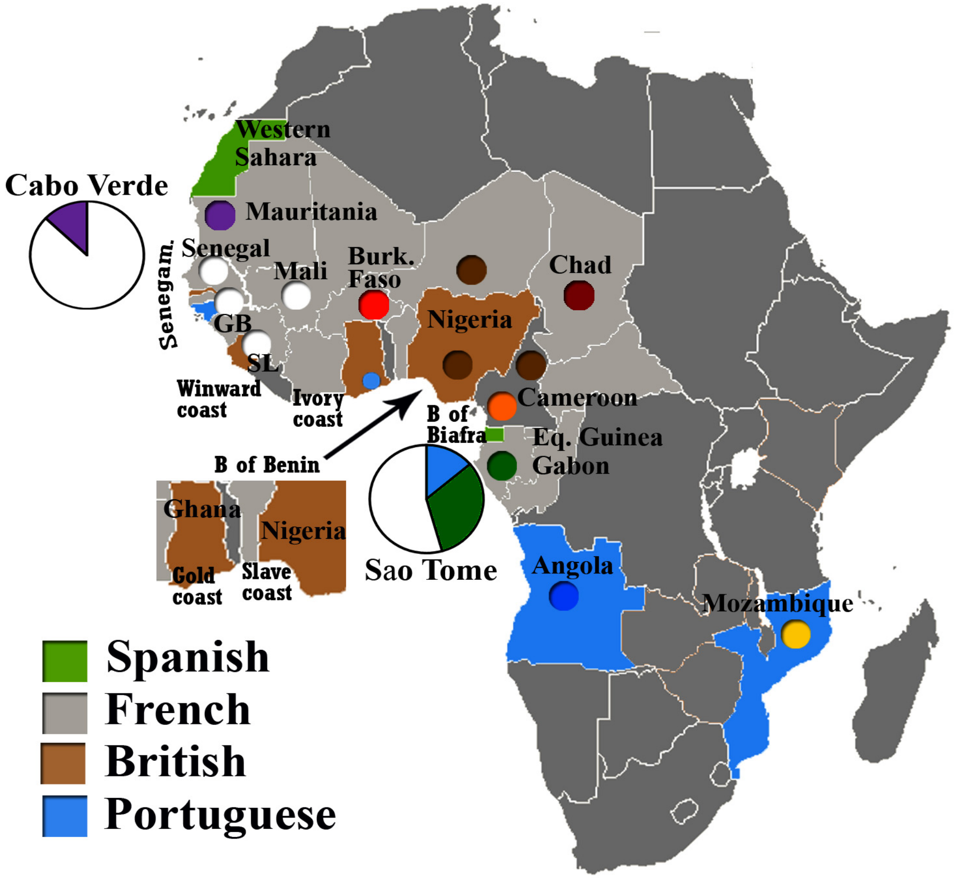

Figure S6: **Map of Africa with source W/WC, SW/WC Bantu, and SE African populations and their contribution to the admixed populations of São Tomé e Príncipe and Cabo Verde.** The pie charts show the relative contribution of mtDNA variation from the sampled populations of W/WC, SW/WC Bantu and SE Africa that founded to the populations of Cabo Verde and São Tomé e Príncipe. The relevant colonies are illustrated on the map along with the major historical coastlines of W/WC Africa matched to the current geography. In Cabo Verde, mainly the neighboring West African populations contributed mtDNA diversity (~87% from Senegambia region and ~13% from Mauritania/Western Sahara), while mtDNA of São Toméans was drawn both from directly neighboring SW/WC Bantu (Equatorial Guinea/Gabon and Angola) and W African populations (Senegambia) (40% vs. 60%).

Table S6: The relative contribution of African regions to the admixed populations of the Americas and Africa, analyzed using ADMIX and reported as a mean ± SD (for details on admixed populations see **Text S1**). Founding African populations include SW/WC Bantu, and SE and W/WC Africa subdivided according to geography. W/WC Africa was also subdivided according to language and combination of ethnicity/language/geography*. Estimates that did not fit our criteria (2SDs >0) yet were high and consistent enough to be considered guidance are indicated in grey (the high SD was due to combination of small sizes of both admixed population and source population) and the alternative relative contribution indicated by asterisk.

	SE	SW/WC Bantu			W/WC-geography			
	Mozambique	Angola	Cameroon	Gabon Eq. Guinea	GuinBis Mali Senegal Sier Leo	Niger Nigeria Cameroon	Maurit WSahara	Burkina Faso
Pops (# of samples)	n=417	n=154	n=359	n=876	n=1133	n=724	n=89	n=97
Sao Tome (n=152)	x	13.9 ± 9.5*	x	31.6 ± 9*	54.5 ± 6*	x	x	x
		x	x	40 ± 6	60 ± 6			
Cabo Verde (n=283)	x	x	x	x	87.2 ± 6	x	12.8 ± 6	x
Brazil (n=404)	13.6 ± 4	32.3 ± 10	x	12.9 ± 5	28.9 ± 6	12.3 ± 6	x	x
Colombia (n=69)	9.6 ± 4	x	x	27.5 ± 8	62.9 ± 7	x	x	x
Caribbean (n=283)	x	24.9 ± 7	x	x	45.9 ± 8	29.2 ± 9	x	x
Cuba (n=112)	x	x	21 ± 7	x	42.2 ± 10	36.7 ± 10	x	x
USA (n=704)	1.5 ± 2.5	17.6 ± 7	14.2 ± 6	x	43.4 ± 6	24.8 ± 7	x	x
					38.6 ± 7*	22.3 ± 7*	x	7.3 ± 3.5*
Philadelphia (n=191)	2.8 ± 5.7	26.2 ± 11	15 ± 7	x	21.7 ± 8	37.2 ± 9	x	x

Table S6 (continued)

	W/WC-language			W/WC-Ethnic groups 1				W/WC-Ethnic groups 2			
	AtlNS+Mande	Volta Chadic Nilo Sah	Berber Semitic	W_Niger Congo	WC_AfAs_Volta	Fulbe Mende	Sem Ber of Mali Murit W Sah	CamNig ChadVolt 1	Lebou Wolof Senegal 2	Sen GB Mali SierLeo 3	Fulbe CamNig BurkFas 4
AA/# of samples	n=1392	n=593	n=181	n=793	n=542	n=457	n=116	n=429	n=93	n=751	n=230
Sao Tome (n=152)	58.2 ± 6	x	x	60.5 ± 6	x	x	x	x<br>28 ± 10*	45.4 ± 8<br>32 ± 10*	x<br>x	x<br>x
Cabo Verde (n=283)	78.5 ± 9	x	21.5 ± 9	82.9 ± 8	x	x	17.1 ± 8	x	41 ± 23*	59 ± 23*	x
Brazil (n=404)	25.7 ± 5	15.5 ± 5	x	30.7 ± 5	10.5 ± 5	x	x	18.6 ± 7	22.6 ± 6	x	x
Colombia (n=69)	41.9 ± 10	x	21 ± 10	40.5 ± 11	12 ± 8	x	10.4 ± 7	x	55.6 ± 9	x	x
Caribbean (n=283)	45.6 ± 7	29.4 ± 7	x	44 ± 9*<br>51.9 ± 7	21 ± 7*<br>23.2 ± 7	11 ±7*<br>x	x	27.1 ± 8	x	48.2 ± 7	x
Cuba (n=112)	47.9 ± 9	30.9 ± 9	x	49.9 ± 9	29 ± 8	x	x	32.2 ± 10	x	46.8 ± 10	x
USA (n=704)	46.2 ± 5	22 ± 6	x	36.7 ± 8	12.7 ± 5.5	18.8 ±6	x	18 ± 6	x	37.8 ± 7	12.4 ± 5
Philadelphia (n=191)	27.4 ± 7	31.5 ± 8	x	20 ± 11*<br>31.9 ± 7	23 ± 7*<br>27 ± 7.5	15 ± 8*<br>x	x	30 ± 9*<br>33.1 ± 9	x<br>x	17 ± 10*<br>25.8 ± 7	11 ± 7*<br>x

*Note: For details on “W/WC-language” and “W/WC-Ethnic groups 1” see text in **Figures S3** and **S4**, respectively. For “W/WC-Ethnic groups 2” the groups are defined as follows (details in **Figure S5** and **Text S1**): Group 1: Chadic and Volta speakers of Cameroon, Niger, and Nigeria (Sonrhei, Songhai, Kanuri, Hide, Podokwo, Masa, Mafa, Uldeme, Fali, Tali, Tupuri, Mandara, Daba, Yoruba); Group 2: Atlantic N speakers of Senegal (Lebou, Wolof, Serer); Group 3: Atlantic N&S and Mande speakers of Guinea Bissau, Senegal, Mali, and Sierra Leone (Brame, Papel, Manjaco, Mankanya, Bijagó, Nalu, Sussu, Peul, Peulh, Poular, Tukulor, Fulbe, Limba, Temne, Bambara, Malinke, Baiote, Diola, Banhu, Cassanga, Beafada, Bainouk, Balanta); and Group 4: Fulbe of Cameroon, Burkina Faso, Nigeria, and Niger (Atlantic N).

Table S7: Relative contribution of African regions to the admixed populations as represented in Figure 3b. The contributions are adapted from **Table S6** (simplified and showing only the American admixed populations).

	SE	SW/WC Bantu			W/WC-geography	
	Mozam bique	Angola	Camer.	Gabon, Eq. Guinea	G.Bis, Mali, Senegal, Sierra Leo.	Niger, Nigeria, Camer.
Brazil	14%	32%	-	13%	29%	12%
Colombia	10%	-	-	27%	63%	-
Caribbean	-	25%	-	-	46%	29%
Cuba	-	-	21%	-	42%	37%
USA	-	18%	14%	-	43%	25%
Philadelphia	-	26%	15%	-	22%	37%

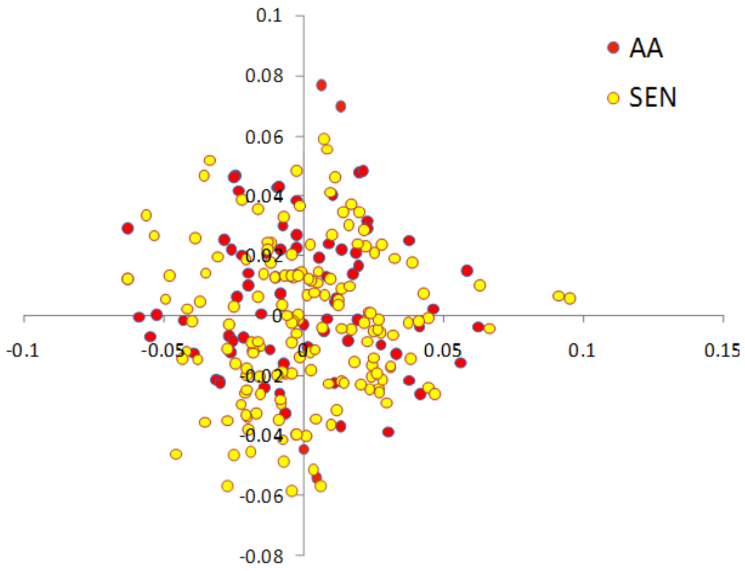

Figure S7: MDS plot of Senegalese and Philadelphia African Americans based on AIMs. MDS plot shows no clustering of Senegalese (yellow) or African Americans from Philadelphia (red, only samples with <5% of European ancestry were included) when AIMs designed to separate European and African ancestry (n=175) were used. Despite Philadelphia African Americans have only ~20% of West African ancestry with the other 80% being primarily drawn from WC/SW Africa, no obvious clustering was observed.

Table S8: LnP(D) for STRUCTURE admixture estimate runs assuming 1-5 populations (K=1-5).

	K=1	K=2	**K=3**	K=4	K=5
LnP(D)	-387 072.7	-269 592.9	**-268 081.5**	-268 674.2	-267 137.4

Text S1: Detailed description of mtDNA database, grouping of African ethnic groups, the historical context for African/American populations, and admixture analysis using ADMIX.

1) Detailed description of mtDNA database

The African part of mtDNA database focused mainly on the West and West-Central parts of Africa, but included all other parts of Africa (North, North-East, South-West, South, East, and most importantly South-East). It includes the following states (the number of sequences in brackets): Angola (154), Botswana (19), Burkina Faso (97), Cameroon (1074), Central African Republic (C.A.R., 130), Chad (124), Democratic Republic of Congo (D.R.C., 62), Egypt (125), Equatorial Guinea (45), Eritrea (8), Ethiopia (262), Gabon (907), Guinea-Bissau (372), Kenya (154), Mali (204), Mauritania (64), Morocco (92), Mozambique (417), Niger (88), Nigeria (171), Senegal (280), Sierra Leone (277), Somalia (27), South Africa (74), Sudan (80), West Sahara (25). The admixed populations included in the database were from: a) USA, namely African Americans from FBI database (n=765), including only the sequences sequenced using R 16,569, except 3 sequences that had various issues; Gullah/Geechee of South Carolina/Georgia (n=78), and Philadelphia (n=217 of African Americans and n=204 of European-Americans), b) Brazil (n=779), divided into Black Brazilians (n=277, with additional n=84 having only partial information), White Brazilians (n=171), and "population of Brazil" (n=247, noted by authors as predominantly white), c) Caribbean (n=313, African-Caribbeans), d) Cuba (n=245, general population), e) Afro-Colombians (n=95), and f) Uruguay (n=43, general population, this dataset was not used in the subsequent analysis because of insufficient sample size), and g) Africa (Cabo Verde, n=291 and São Tomé e Príncipe, n=153). While building the database, 16,182C and 16,183C (mostly linked to 16,189C mutation) were omitted because of inconsistent reporting among the publications. Further, mutation in position 16,390 was added when the sequencing primers ended before this position but the authors checked for L2 status using RFLP. The variation in the database was then collapsed into 429 distinct haplotypes that were defined while considering the variation within the database.

2) Cluster separation of African variation based on ethnic groups. Our database contains over 65 ethnicities in W/WC/NW/C region (after excluding Bantu and Pygmy). Most of these have insufficient sample size to accurately represent the mtDNA variation within each ethnicity. Since there is no unambiguous way of combining ethnic groups with small sample sizes, we approached this

problem from two directions. First, we identified ethnic groups with >45 samples (except Kanuri - an analysis outlier with small sample size) and grouped all other ethnicities into additional 7 groups, based on geography, language and reported origin (**Table S5**). These 19 groups were analyzed by SAMOVA and divided into 5 clusters: 1) WC speakers of Afro-Asiatic and Volta-Congo languages (comprised of Arabs, Chadic speakers of Cameroon, including eastern origin Fali, Kanuri, and a group of Volta speakers), 2) W speakers of Niger-Congo (including Atlantic North or Mande and single ethnic groups including Balanta, Bambara, Malinke, Limba, Wolof), 3) Mandenka, 4) Fulbe and Mende, and 5) Afro-Asiatic speakers of NW (v_A=2.55%, v_B=0.74%, **Figure S4**). Second, we combined those ethnicities that belong into the same language group, collapsing the total data into 26 distinct groups of populations (**File S5**). Using the same SAMOVA grouping method and after excluding 4 outlying groups with small size, the remaining 22 groups can be reduced to 9 clusters that further need to be collapsed for the purpose of admixture analysis into 6, combining small and similar populations (9 groups: v_A=2.43%, v_B^*=0.14%; 6 groups: v_A=2.28%, v_B=0.46%, where genetically similar groups with n<90 were grouped; **Figure S5**). Notable difference was found in the Fulbe people of WC and W that were grouped in the previous analysis. Also, two new groups were separated from the West Niger-Congo cluster: Loko-Mende of Sierra Leone and Lebou-Wolof-Serer of Senegal. As discussed below, these groupings provide us with higher resolution in admixture analysis for some of the admixed populations.

3) The historical context of regions in Africa and sampled admixed populations.

The historical context of regions in Africa and sampled admixed populations from former colonies: 1) *Africa*: a) **SE**: Mozambique (Portuguese), Kenya (British); b) **SW/WC**: Angola/Cabinda (Portuguese), Gabon/Equatorial Guinea (Spanish/French), Cameroon (German, bordering British Nigeria); c) **W/WC**: Senegal + Mali (French)/Gambia + Sierra Leone (British)/Guinea Bissau (Portuguese) and Nigeria (British, part of the neighboring coast Portuguese)/Cameroon (German) and the Berber/Semitic speakers drawn mainly from Spanish West Sahara, French Mauritania and British Nigeria; 2) *Americas:* Philadelphia (British), USA (British, French, Spanish), Caribbean (British, Spanish, French, and other), Colombia (Spanish), Brazil (Portuguese).

4) Detail description of admixture analysis using ADMIX. The following datasets were used for calculating the admixture proportions for different populations, ignoring (or indicating in grey in **Table S6**) the contribution from founding populations whose admixture coefficients were outside 2 bootstrap standard deviations of 0:

a) **European and Native American admixture reflected in mtDNA pool of African Americans:** In order to establish the contribution of European, Native American and African populations to the primarily African admixed populations: African Brazilians (Braz_AA, n=277), Afro-Colombians (Col_AA, n=95), Afro-Caribbeans (Carib_AA, n=313), African Americans from Philadelphia (Phila_AA, n=217) or South Carolina/Georgia (Gul_AA, n=78) or the whole USA (USA_AA, n=765), we have used Europe combined with Eurasia, Native American, W/WC non-Bantu/non-Pygmy, Bantu of WC/SW, and SE African datasets as the parental populations. The African proportions were established as a sum of contributions from all three African regions.

b) **Within-Africa mtDNA origin of American admixed populations**: We have estimated the contribution of W/WC non-Bantu/non-Pygmy, Bantu of WC/SW, and SE Africa to the primarily African admixed populations Braz_AA (n=404), Col_AA (n=79), Carib_AA (n=283), Phila_AA (n=191), USA_AA (n=704), as well as populations of mixed ancestry: Cuba (n=112), considering only the African-derived haplogroups (L, U6, U5b1b) for the admixed populations. We have further attempted to subdivide the contribution from W/WC non-Bantu/non-Pygmy, Bantu of SW/WC, and SE Africa into the predefined regions: 1) W/WC non-Bantu/non-Pygmy was separated according to geography (**Figure S3**), language (**Figure S4**), and ethnicity/geography/language (**Figure S5 and S6**), 2) Bantu of WC/SW was separated according to geography into Bantu of Cameroon, Equatorial Guinea and Gabon, and Angola, and 3) only Mozambique (as the more probable source of variation) in SE Africa was considered (also based on the fact that the calculated contribution from Kenya was found to be minimal).

References S1:

1. Allard MW, Polanskey D, Miller K, Wilson MR, Monson KL, et al. (2005) Characterization of human control region sequences of the African American SWGDAM forensic mtDNA data set. Forensic Sci Int 148: 169-179.
2. Alves-Silva J, da Silva Santos M, Guimaraes PE, Ferreira AC, Bandelt HJ, et al. (2000) The ancestry of Brazilian mtDNA lineages. Am J Hum Genet 67: 444-461.

3. Benn Torres J, Kittles RA, Stone AC (2007) Mitochondrial and Y chromosome diversity in the English-speaking Caribbean. Ann Hum Genet 71: 782-790.
4. Ely B, Wilson JL, Jackson F, Jackson BA (2006) African-American mitochondrial DNAs often match mtDNAs found in multiple African ethnic groups. BMC Biol 4: 34.
5. Goncalves VF, Carvalho CM, Bortolini MC, Bydlowski SP, Pena SD (2008) The phylogeography of African Brazilians. Hum Hered 65: 23-32.
6. Guerreiro V, Bisso-Machado R, Marrero A, Hunemeier T, Salzano FM, et al. (2009) Genetic signatures of parental contribution in black and white populations in Brazil. Genetics and Molecular Biology 32: 1-11.
7. Hunemeier T, Carvalho C, Marrero AR, Salzano FM, Junho Pena SD, et al. (2007) Niger-Congo speaking populations and the formation of the Brazilian gene pool: mtDNA and Y-chromosome data. Am J Phys Anthropol 133: 854-867.
8. Mendizabal I, Sandoval K, Berniell-Lee G, Calafell F, Salas A, et al. (2008) Genetic origin, admixture, and asymmetry in maternal and paternal human lineages in Cuba. BMC Evol Biol 8: 213.
9. Salas A, Acosta A, Alvarez-Iglesias V, Cerezo M, Phillips C, et al. (2008) The mtDNA ancestry of admixed Colombian populations. Am J Hum Biol 20: 584-591.
10. Sans M, Weimer TA, Franco MH, Salzano FM, Bentancor N, et al. (2002) Unequal contributions of male and female gene pools from parental populations in the African descendants of the city of Melo, Uruguay. Am J Phys Anthropol 118: 33-44.
11. Silva WA, Bortolini MC, Schneider MP, Marrero A, Elion J, et al. (2006) MtDNA haplogroup analysis of black Brazilian and sub-Saharan populations: implications for the Atlantic slave trade. Hum Biol 78: 29-41.
12. Stefflova K, Dulik MC, Pai AA, Walker AH, Zeigler-Johnson C, et al. (2009) Evaluation of Group Genetic Ancestry of Populations from Philadelphia and Dakar in the Context of Sex-biased Admixture in the Americas. Accepted in PLoS ONE.
13. Beleza S, Gusmao L, Amorim A, Carracedo A, Salas A (2005) The genetic legacy of western Bantu migrations. Hum Genet 117: 366-375.
14. Brandstatter A, Peterson CT, Irwin JA, Mpoke S, Koech DK, et al. (2004) Mitochondrial DNA control region sequences from Nairobi (Kenya): inferring phylogenetic parameters for the establishment of a forensic database. Int J Legal Med 118: 294-306.
15. Brehm A, Pereira L, Bandelt HJ, Prata MJ, Amorim A (2002) Mitochondrial portrait of the Cabo Verde archipelago: the Senegambian outpost of Atlantic slave trade. Ann Hum Genet 66: 49-60.
16. Cerny V, Hajek M, Cmejla R, Bruzek J, Brdicka R (2004) mtDNA sequences of Chadic-speaking populations from northern Cameroon suggest their affinities with eastern Africa. Ann Hum Biol 31: 554-569.
17. Cerny V, Hajek M, Bromova M, Cmejla R, Diallo I, et al. (2006) MtDNA of Fulani nomads and their genetic relationships to neighboring sedentary populations. Hum Biol 78: 9-27.
18. Cerny V, Salas A, Hajek M, Zaloudkova M, Brdicka R (2007) A bidirectional corridor in the Sahel-Sudan belt and the distinctive features of the Chad Basin populations: a history revealed by the mitochondrial DNA genome. Ann Hum Genet 71: 433-452.
19. Chen YS, Olckers A, Schurr TG, Kogelnik AM, Huoponen K, et al. (2000) mtDNA variation in the South African Kung and Khwe- and their genetic relationships to other African populations. Am J Hum Genet 66: 1362-1383.

20. Coia V, Destro-Bisol G, Verginelli F, Battaggia C, Boschi I, et al. (2005) Brief communication: mtDNA variation in North Cameroon: lack of Asian lineages and implications for back migration from Asia to sub-Saharan Africa. Am J Phys Anthropol 128: 678-681.
21. Destro-Bisol G, Coia V, Boschi I, Verginelli F, Caglia A, et al. (2004) The analysis of variation of mtDNA hypervariable region 1 suggests that Eastern and Western Pygmies diverged before the Bantu expansion. Am Nat 163: 212-226.
22. Gonzalez AM, Cabrera VM, Larruga JM, Tounkara A, Noumsi G, et al. (2006) Mitochondrial DNA variation in Mauritania and Mali and their genetic relationship to other Western Africa populations. Ann Hum Genet 70: 631-657.
23. Jackson BA, Wilson JL, Kirbah S, Sidney SS, Rosenberger J, et al. (2005) Mitochondrial DNA genetic diversity among four ethnic groups in Sierra Leone. Am J Phys Anthropol 128: 156-163.
24. Kivisild T, Reidla M, Metspalu E, Rosa A, Brehm A, et al. (2004) Ethiopian mitochondrial DNA heritage: tracking gene flow across and around the gate of tears. Am J Hum Genet 75: 752-770.
25. Mateu E, Comas D, Calafell F, Perez-Lezaun A, Abade A, et al. (1997) A tale of two islands: population history and mitochondrial DNA sequence variation of Bioko and Sao Tome, Gulf of Guinea. Ann Hum Genet 61: 507-518.
26. Pereira L, Macaulay V, Torroni A, Scozzari R, Prata MJ, et al. (2001) Prehistoric and historic traces in the mtDNA of Mozambique: insights into the Bantu expansions and the slave trade. Ann Hum Genet 65: 439-458.
27. Plaza S, Salas A, Calafell F, Corte-Real F, Bertranpetit J, et al. (2004) Insights into the western Bantu dispersal: mtDNA lineage analysis in Angola. Hum Genet 115: 439-447.
28. Quintana-Murci L, Quach H, Harmant C, Luca F, Massonnet B, et al. (2008) Maternal traces of deep common ancestry and asymmetric gene flow between Pygmy hunter-gatherers and Bantu-speaking farmers. Proc Natl Acad Sci U S A 105: 1596-1601.
29. Rando JC, Pinto F, Gonzalez AM, Hernandez M, Larruga JM, et al. (1998) Mitochondrial DNA analysis of northwest African populations reveals genetic exchanges with European, near-eastern, and sub-Saharan populations. Ann Hum Genet 62: 531-550.
30. Richards M, Macaulay V, Hickey E, Vega E, Sykes B, et al. (2000) Tracing European founder lineages in the Near Eastern mtDNA pool. Am J Hum Genet 67: 1251-1276.
31. Rosa A, Brehm A, Kivisild T, Metspalu E, Villems R (2004) MtDNA profile of West Africa Guineans: towards a better understanding of the Senegambia region. Ann Hum Genet 68: 340-352.
32. Salas A, Richards M, De la Fe T, Lareu MV, Sobrino B, et al. (2002) The making of the African mtDNA landscape. Am J Hum Genet 71: 1082-1111.
33. Stevanovitch A, Gilles A, Bouzaid E, Kefi R, Paris F, et al. (2004) Mitochondrial DNA sequence diversity in a sedentary population from Egypt. Ann Hum Genet 68: 23-39.
34. Trovoada MJ, Pereira L, Gusmao L, Abade A, Amorim A, et al. (2004) Pattern of mtDNA variation in three populations from Sao Tome e Principe. Ann Hum Genet 68: 40-54.
35. Watson E, Forster P, Richards M, Bandelt HJ (1997) Mitochondrial footprints of human expansions in Africa. Am J Hum Genet 61: 691-704.
36. Fagundes NJ, Kanitz R, Eckert R, Valls AC, Bogo MR, et al. (2008) Mitochondrial population genomics supports a single pre-Clovis origin with a coastal route for the peopling of the Americas. Am J Hum Genet 82: 583-592.

37. Li H, Cai X, Winograd-Cort ER, Wen B, Cheng X, et al. (2007) Mitochondrial DNA diversity and population differentiation in southern East Asia. Am J Phys Anthropol 134: 481-488.
38. Malyarchuk BA, Grzybowski T, Derenko MV, Czarny J, Drobnic K, et al. (2003) Mitochondrial DNA variability in Bosnians and Slovenians. Ann Hum Genet 67: 412-425.
39. Brion M, Sanchez JJ, Balogh K, Thacker C, Blanco-Verea A, et al. (2005) Introduction of an single nucleodite polymorphism-based "Major Y-chromosome haplogroup typing kit" suitable for predicting the geographical origin of male lineages. Electrophoresis 26: 4411-4420.
40. Hammer MF, Chamberlain VF, Kearney VF, Stover D, Zhang G, et al. (2006) Population structure of Y chromosome SNP haplogroups in the United States and forensic implications for constructing Y chromosome STR databases. Forensic Sci Int 164: 45-55.
41. Silva DA, Carvalho E, Costa G, Tavares L, Amorim A, et al. (2006) Y-chromosome genetic variation in Rio de Janeiro population. Am J Hum Biol 18: 829-837.
42. Vallone PM, Butler JM (2004) Y-SNP typing of U.S. African American and Caucasian samples using allele-specific hybridization and primer extension. J Forensic Sci 49: 723-732.
43. Goncalves R, Freitas A, Branco M, Rosa A, Fernandes AT, et al. (2005) Y-chromosome lineages from Portugal, Madeira and Acores record elements of Sephardim and Berber ancestry. Ann Hum Genet 69: 443-454.
44. Goncalves R, Spinola H, Brehm A (2007) Y-chromosome lineages in Sao Tome e Principe islands: evidence of European influence. Am J Hum Biol 19: 422-428.
45. Cruciani F, Santolamazza P, Shen P, Macaulay V, Moral P, et al. (2002) A back migration from Asia to sub-Saharan Africa is supported by high-resolution analysis of human Y-chromosome haplotypes. Am J Hum Genet 70: 1197-1214.
46. Luis JR, Rowold DJ, Regueiro M, Caeiro B, Cinnioglu C, et al. (2004) The Levant versus the Horn of Africa: evidence for bidirectional corridors of human migrations. Am J Hum Genet 74: 532-544.
47. Robino C, Crobu F, Di Gaetano C, Bekada A, Benhamamouch S, et al. (2008) Analysis of Y-chromosomal SNP haplogroups and STR haplotypes in an Algerian population sample. Int J Legal Med 122: 251-255.
48. Rosa A, Ornelas C, Jobling MA, Brehm A, Villems R (2007) Y-chromosomal diversity in the population of Guinea-Bissau: a multiethnic perspective. BMC Evol Biol 7: 124.
49. Tishkoff SA, Gonder MK, Henn BM, Mortensen H, Knight A, et al. (2007) History of click-speaking populations of Africa inferred from mtDNA and Y chromosome genetic variation. Mol Biol Evol 24: 2180-2195.
50. Wood ET, Stover DA, Ehret C, Destro-Bisol G, Spedini G, et al. (2005) Contrasting patterns of Y chromosome and mtDNA variation in Africa: evidence for sex-biased demographic processes. Eur J Hum Genet 13: 867-876.
51. Yaeger R, Avila-Bront A, Abdul K, Nolan PC, Grann VR, et al. (2008) Comparing genetic ancestry and self-described race in african americans born in the United States and in Africa. Cancer Epidemiol Biomarkers Prev 17: 1329-1338.
52. Benn-Torres J, Bonilla C, Robbins CM, Waterman L, Moses TY, et al. (2008) Admixture and population stratification in African Caribbean populations. Ann Hum Genet 72: 90-98.
53. Salzano FM, Bortolini MC (2002) Evolution and Genetics of Latin American Populations. . Cambridge: Cambridge University Press.
